# Desymmetrizing Heteroleptic [Cu(P^P)(N^N)][PF_6_] Compounds: Effects on Structural and Photophysical Properties, and Solution Dynamic Behavior

**DOI:** 10.3390/molecules26010125

**Published:** 2020-12-29

**Authors:** Marco Meyer, Fabian Brunner, Alessandro Prescimone, Edwin C. Constable, Catherine E. Housecroft

**Affiliations:** Department of Chemistry, University of Basel, BPR 1096, Mattenstrasse 24a, CH-4058 Basel, Switzerland; marco.meyer@unibas.ch (M.M.); fabian.brunner@gmx.de (F.B.); alessandro.prescimone@unibas.ch (A.P.); edwin.constable@unibas.ch (E.C.C.)

**Keywords:** copper(I), bisphosphane, 2,2′-bipyridine, photophysics, X-ray diffraction

## Abstract

The preparation, characterization and electrochemical and photophysical properties of a series of desymmetrized heteroleptic [Cu(P^P)(N^N)][PF_6_] compounds are reported. The complexes incorporate the chelating P^P ligands bis(2-(diphenylphosphanyl)phenyl)ether (POP) and (9,9-dimethyl-9*H*-xanthene-4,5-diyl)bis(diphenylphosphane) (xantphos), and 6-substituted 2,2′-bipyridine (bpy) derivatives with functional groups attached by –(CH_2_)*_n_*– spacers: 6-(2,2′-bipyridin-6-yl)hexanoic acid (**1**), 6-(5-phenylpentyl)-2,2′-bipyridine (**2**) and 6-[2-(4-phenyl-1*H*-1,2,3,triazol-1-yl)ethyl]-2,2′-bipyridine (**3**). [Cu(POP)(**1**)][PF_6_], [Cu(xantphos)(**1**)][PF_6_], [Cu(POP)(**2**)][PF_6_], [Cu(xantphos)(**2**)][PF_6_], and [Cu(xantphos)(**3**)][PF_6_] have been characterized in solution using multinuclear NMR spectroscopy, and the single crystal structure of [Cu(xantphos)(**3**)][PF_6_]**^.^**0.5Et_2_O was determined. The conformation of the 6-[2-(4-phenyl-1*H*-1,2,3,triazol-1-yl)ethyl]-substituent in the [Cu(xantphos)(**3**)]^+^ cation is such that the α- and β-CH_2_ units reside in the xanthene ‘bowl’ of the xantphos ligand. The 6-substituent desymmetrizes the structure of the [Cu(P^P)(N^N)]^+^ cation and this has consequences for the interpretation of the solution NMR spectra of the five complexes. The NOESY spectra and EXSY cross-peaks provide insight into the dynamic processes operating in the different compounds. For powdered samples, emission maxima are in the range 542–555 nm and photoluminescence quantum yields (PLQYs) lie in the range 13–28%, and a comparison of PLQYs and decay lifetimes with those of [Cu(xantphos)(6-Mebpy)][PF_6_] indicate that the introduction of the 6-substituent is not detrimental in terms of the photophysical properties.

## 1. Introduction

Copper(I) coordination compounds are a focus of attention as efficient emissive materials for organic light emitting diodes (OLEDs) [1] and light-emitting electrochemical cells (LECs) [2,3,4,5,6]. In a LEC, an ionic transition metal complex (iTMC) can function both as the emitter and as charge carrier. Heteroleptic [Cu(P^P)(N^N)]^+^ complexes in which N^N is a diimine and P^P is a bisphosphane are of particular interest because many exhibit thermally activated delayed fluorescence (TADF) [7,8,9]. In TADF-compounds, the energy gap between the excited singlet and triplet states is small, leading to reverse intersystem crossing which gives rise to enhanced photoluminescence quantum yields (PLQYs). The current generations of [Cu(P^P)(N^N)]^+^ complexes have their origins in derivatives containing 2,2′-bipyridine (bpy) or 1,10-phenanthroline (phen) which were found by McMillin to exhibit low-lying metal-to-ligand charge transfer (MLCT) excited states [10,11].

**Scheme 1:**
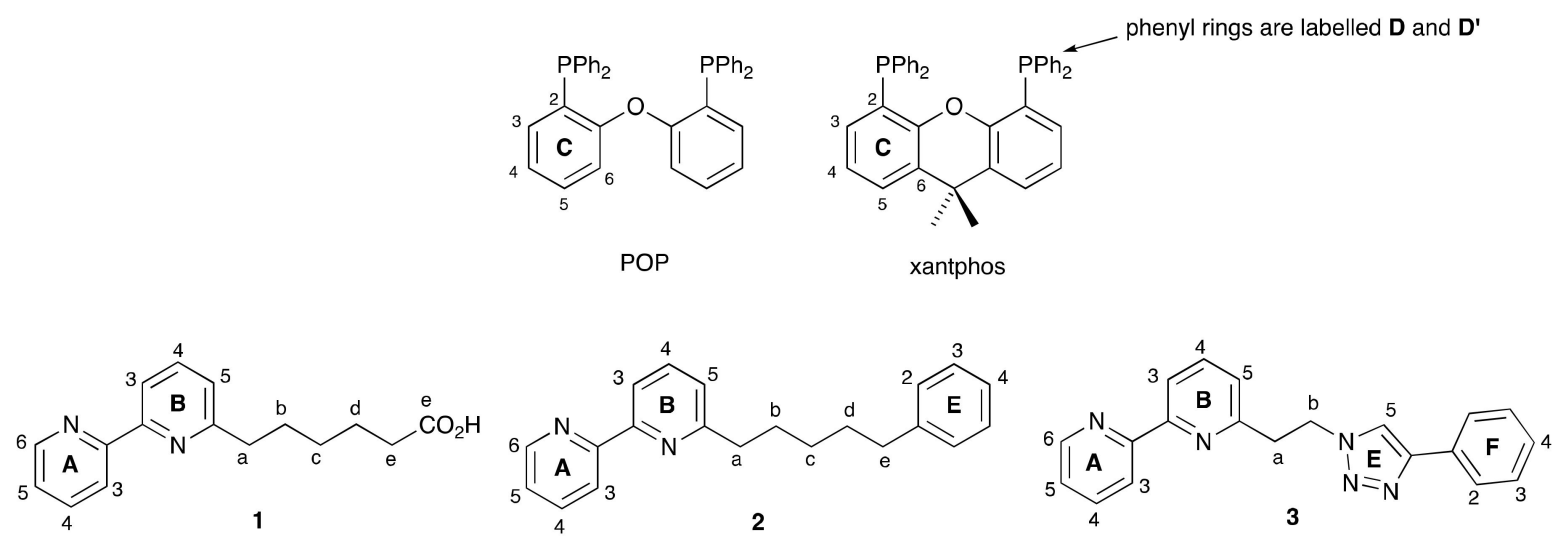
Structures of the P^P and N^N ligands with labelling for the NMR assignments.

Improving the photophysical properties of [Cu(P^P)(N^N)]^+^ complexes can be approached by structural modification of either the P^P or N^N domains. Typically, the P^P ligand is a wide bite-angle bisphosphane such as bis(2-(diphenylphosphanyl)phenyl)ether (POP) or (9,9-dimethyl-9*H*-xanthene-4,5-diyl)bis(diphenylphosphane) (xantphos) (Scheme 1) and these commercially available ligands remain the most popular choices. While structure–property relationships may be developed [12,13,14], enhanced PLQY is most often the result of trial-and-error structural variation of the N^N ligand. Synthetically, it is easier to vary the functionalities in the latter than in the P^P domain. It has also been shown that intramolecular π-stacking interactions in [Cu(P^P)(phen)]^+^ and [Cu(P^P)(4,7-Ph_2_phen)]^+^ (4,7-Ph_2_phen = 4,7-diphenyl-1,10-phenanthroline) lead to increased PLQY values because of inhibition of the flattening of the coordination sphere in the excited state [15]. In addition to enhancing photophysical behaviour, one of the challenges in the design of [Cu(P^P)(N^N)]^+^ emitters is to minimize the tendency for ligand-redistribution reactions. One approach has been to use macrocyclic ligands to produce pseudorotaxanes [16]. The covalent linkage of the P^P and N^N domains is an attractive way forward but appears to have been little explored. This approach will involve the use of longer chains as linkers, and strategies that might be developed involve condensation reactions between appropriate functionalities on the P^P and N^N domains, or the use of click chemistry. In this paper, we consider the consequences on the structural, dynamic and photophysical properties of copper(I) iTMCs of the desymmetrization caused by the introduction of model linker chains at the 6-position of a bpy (ligands **1**–**3** in Scheme 1). We have previously demonstrated that the introduction of 6-methyl, 6-ethyl, 6-phenyl or 6-phenylthio substituents is beneficial to the photophysical properties of [Cu(POP)(bpy)]^+^ and [Cu(xantphos)(bpy)]^+^ derivatives [17,18,19]. However, to the best of our knowledge, little is known about the effects of introducing longer, and potentially sterically demanding, 6-substituents.

## 2. Results and Discussion

### 2.1. Ligand Syntheses

**Scheme 2:**
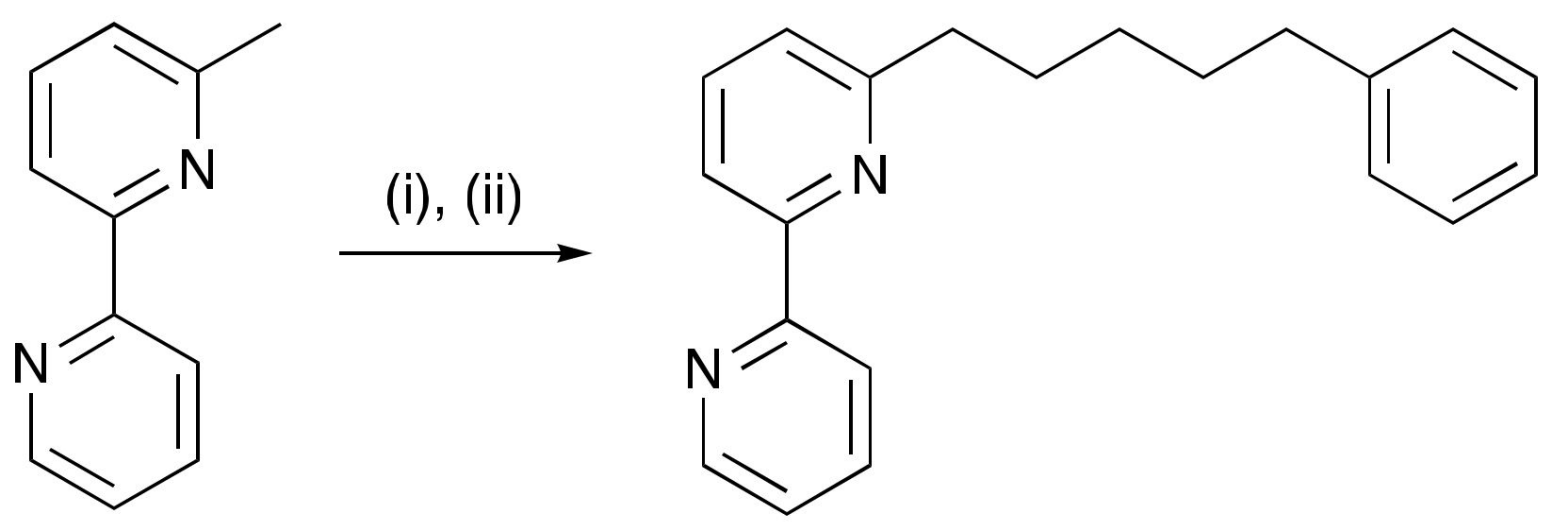
Synthetic route to compound **2**. Reagents and Conditions: (i) Lithium diisopropylamide (LDA), THF, −78 °C; (ii) 1-bromo-4-phenylbutane, THF, room temperature. Yield = 30%.

Compound **1** has previously been reported and was prepared according to the published method [20]. The route shown in Scheme 2 was used to synthesize compound **2**. The first step was lithiation of the methyl group in 6-methyl-2,2′-bipyridine (6-Mebpy) using lithium diisopropylamide (LDA) prepared in situ. The intermediate was then treated with 1-bromo-4-phenylbutane to give **2**. The base peak at *m*/*z* 303.10 in the electrospray (ESI) mass spectrum arose from the [M + H]^+^ ion. The ^1^H- and ^13^C{^1^H}-NMR spectra were assigned using 2D methods, and HMQC and HMBC spectra are shown in Figures S1 and S2 in the Supplementary Materials.

**Scheme 3:**
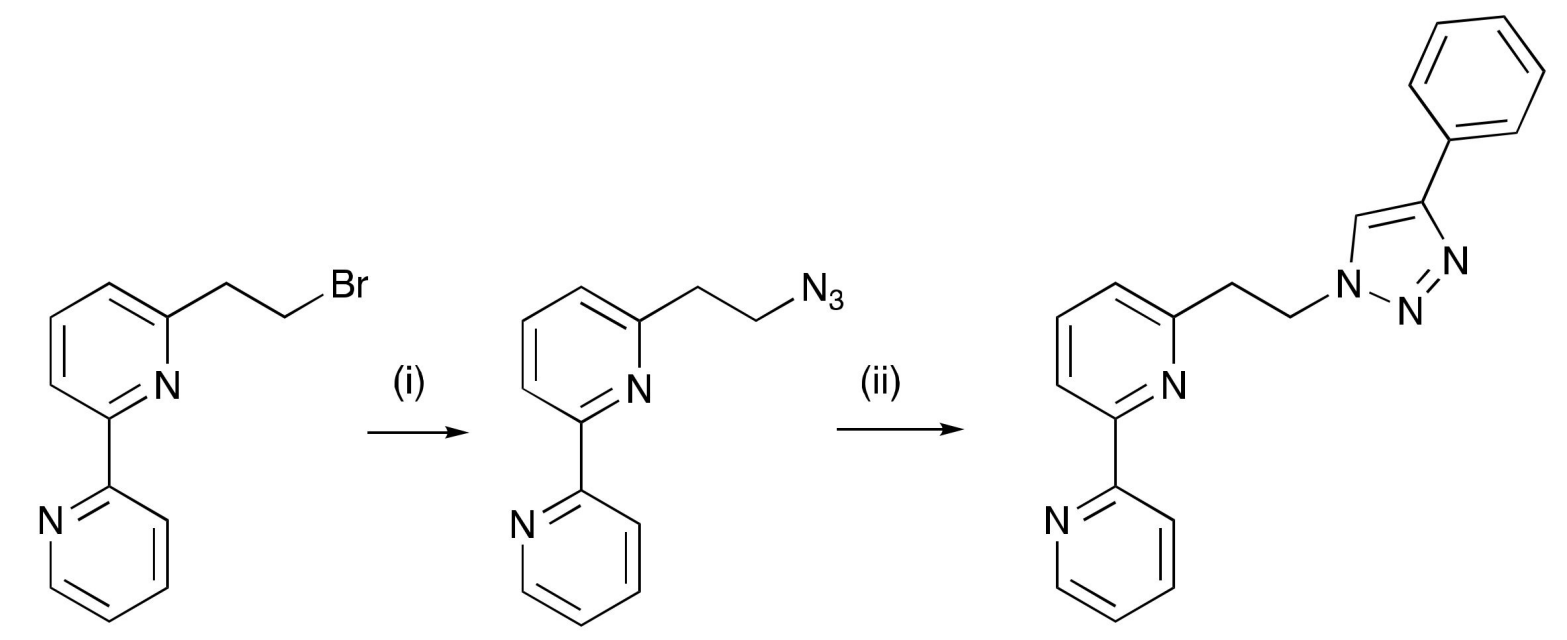
Synthetic route to compound **3**. Reagents and Conditions: (i) NaN_3_, DMF, room temperature; (ii) phenylethyne, CuSO_4_**^.^**5H_2_O, sodium ascorbate, *t*-BuOH/H_2_O, 35 °C. Yield = 54%.

**Figure 1:**
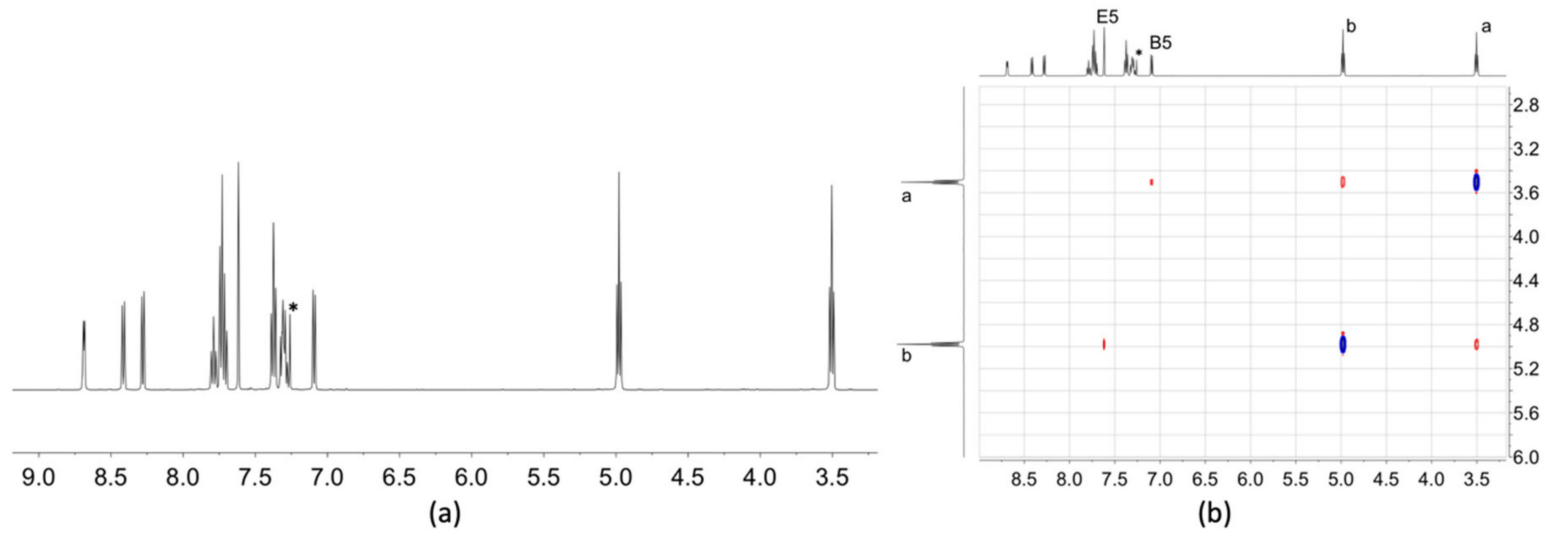
(**a**) The ^1^H-NMR spectrum (500 MHz, CDCl_3_, 298 K) of compound **3**, and (**b**) part of the NOESY spectrum of **3** (500 MHz, CDCl_3_, 298 K). * = residual CHCl_3_.

Compound **3** was prepared by adapting a literature procedure [21] and the synthetic route is summarized in Scheme 3. The base peak (*m*/*z* 328.08) in the ESI mass spectrum corresponded to the [M + H]^+^ ion, and the ^1^H and ^13^C{^1^H} NMR spectra (assigned using COSY, NOESY and HMQC and HMBC methods) were fully in accord with the structure shown in Scheme 3. Figure 1 illustrates the ^1^H NMR spectrum of **3**, and additional spectra are presented in Figures S3 and S4. Using the atom numbering given in Scheme 1, protons H^a^ and H^b^ were distinguished by the observation of NOESY crosspeaks between H^a^ and H^B5^, and between H^b^ and H^E5^ (Figure 1b). 

### 2.2. Synthesis and Characterization of Heteroleptic Copper(I) Complexes

[Cu(POP)(**1**)][PF_6_] and [Cu(POP)(**2**)][PF_6_] were prepared by initially reacting [Cu(MeCN)_4_][PF_6_] and POP in CH_2_Cl_2_, followed by the addition of **1** or **2**. For the xantphos-containing compounds, xantphos and the N^N ligand were added at the same time to a CH_2_Cl_2_ solution of [Cu(MeCN)_4_][PF_6_]. The reasons for these different routes have previously been discussed [18]. [Cu(POP)(**1**)][PF_6_], [Cu(POP)(**2**)][PF_6_], [Cu(xantphos)(**1**)][PF_6_], [Cu(xantphos)(**2**)][PF_6_] and [Cu(xantphos)(**3**)][PF_6_] were isolated as yellow solids in yields ranging from 78% to 99%. Positive-mode ESI mass spectra showed peaks arising from the [M − PF_6_]^+^ ion and in all but [Cu(xantphos)(**3**)][PF_6_], this was the base peak. For [Cu(xantphos)(**3**)][PF_6_], a low intensity peak at *m*/*z* 968.26 corresponded to [M − PF_6_]^+^ and the base peak at *m*/*z* 811.16 was assigned to the fragment ion [Cu(xantphos)(Mebpy)]^+^. For each compound, the presence of the [PF_6_]^−^ counterion was confirmed in the negative-mode ESI mass spectrum, and by a characteristic septet in the ^31^P{^1^H} NMR spectrum. A singlet at δ –13.1 ppm in the ^31^P{^1^H} NMR spectra of [Cu(P^P)(**1**)][PF_6_] and [Cu(P^P)(**2**)][PF_6_] was assigned to the POP or xantphos ligand, while in [Cu(xantphos)(**3**)][PF_6_], the xantphos ligand gave rise to a signal at δ –12.7 ppm. Before considering the NMR characterization of the complexes, we present the crystal structure of [Cu(xantphos)(**3**)][PF_6_]**^.^**0.5Et_2_O.

**Figure 2:**
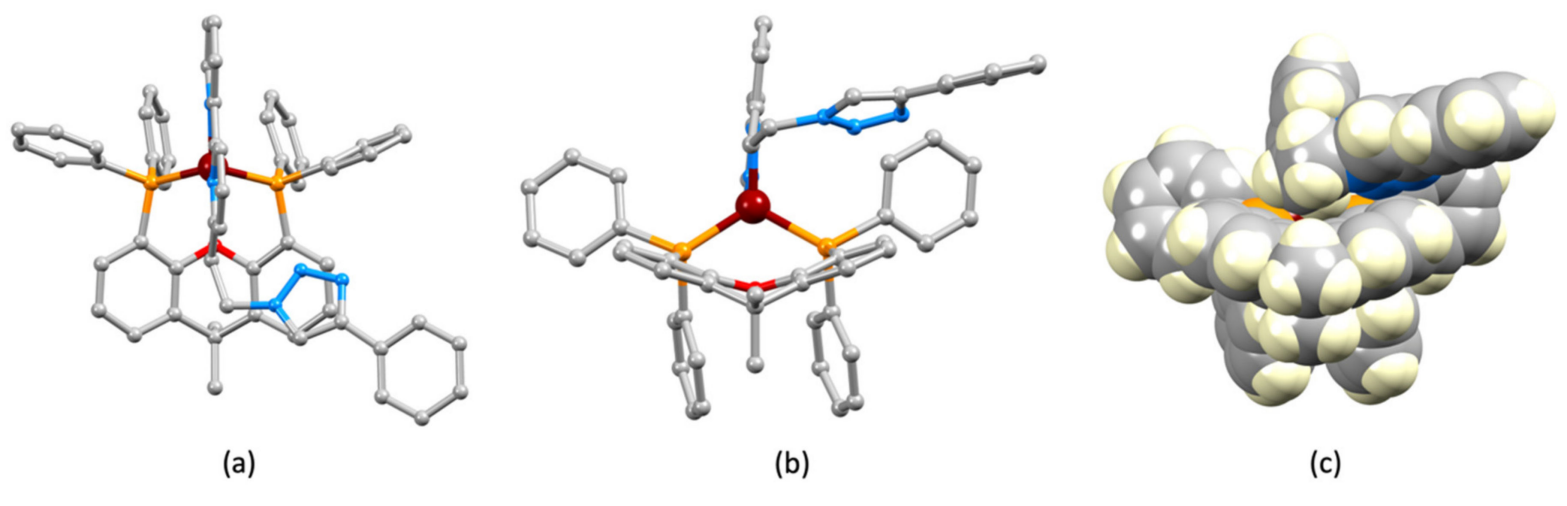
(**a**,**b**) Two views of the [Cu(xantphos)(**3**)]^+^ cation, and (**c**) a space-filling representation of diagram (**b**).

Single crystals of [Cu(xantphos)(**3**)][PF_6_]**^.^**0.5Et_2_O were grown from a CH_2_Cl_2_ of the compound layered with diethyl ether. The compound crystallizes in the triclinic space group *P*–1. Disorder of the phenyl ring of the coordinated ligand **3** (see the experimental section) meant that this part of the structure was refined isotropically, as seen in the ORTEP representation in Figure S5 in the Supplementary Materials. Table 1 presents important bond lengths and angles, and P–C bond lengths are typical, lying in the range 1.827(3) to 1.834(3) Å. Atom Cu1 in the [Cu(xantphos)(**3**)]^+^ cation is in a distorted tetrahedral environment, with the largest angle in the coordination sphere being P2–Cu1–N2 = 118.78(7)^o^. Figure 2a,b show two views of the [Cu(xantphos)(**3**)]^+^ cation, and is noteworthy that the 6-substituent lies over and to one side of the ‘bowl’ shaped cavity of the xanthene; this is relevant to the subsequent NMR spectroscopic discussion. The space-filling diagram in Figure 2c illustrates that the conformation of the chain is such that is desymmetrizes the structure. It is tempting to suggest that this is associated with a stacking of the triazole ring over one arene ring of the xanthene. However, the distance between the ring-centroids is 4.07 Å indicating that this is, at best, a weak π-interaction. Figure 2b reveals that two of the PPh_2_ phenyl rings are aligned to give a π-stacking interaction. The angle between the ring planes is 10.0°, and the centroid...centroid distance is 3.85 Å, making this an effective interaction and one that is typical in many [Cu(xantphos)(N^N)]^+^ complexes [19]. Figure S6 illustrates this interaction and also highlights the proximity of bpy proton H^A6^ (see Scheme 1) to the stacked rings which is pertinent to the NMR spectroscopic discussion below.

**Figure 3:**
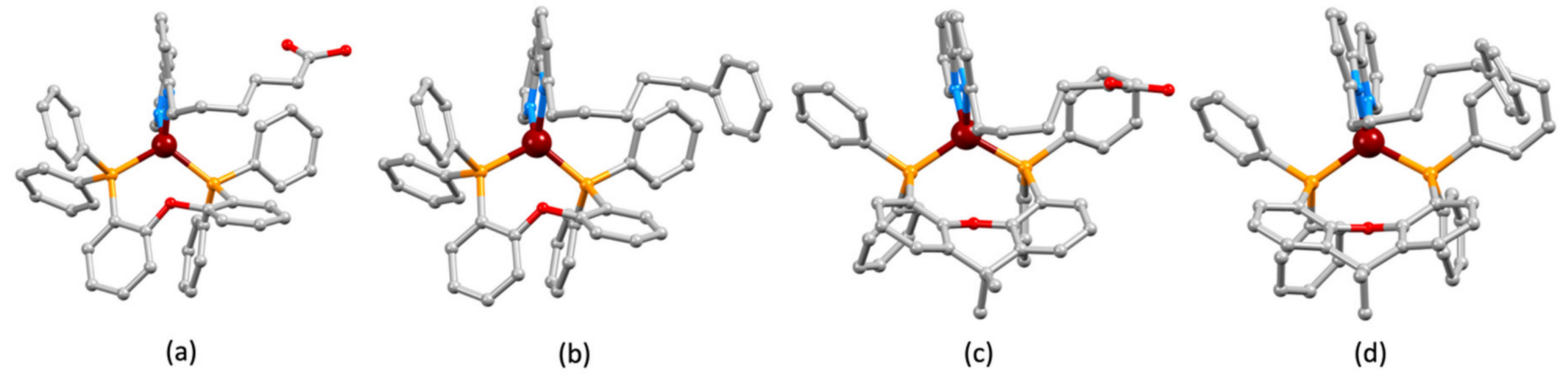
The energy minimized structures of the cations (**a**) [Cu(POP)(**1**)]^+^, (**b**) [Cu(POP)(**2**)]^+^, (**c**) [Cu(xantphos)(**1**)]^+^ and (**d**) [Cu(xantphos)(**2**)]^+^.

Attempts to grow X-ray quality crystals of the remaining four complexes were unsuccessful, and we therefore modelled one of the POP-containing complexes in order to gain information about the structural relationship between the substituent attached to the bpy domain, and the POP ligand. The structure of [Cu(POP)(**1**)]^+^ was minimized, first at a molecular mechanics (MM2) level, and this geometry was used as an input to a DFT level energy minimization (B3LYP 6-31G*) [22]. Figure 3 shows the minimized structures of the [Cu(POP)(**1**)]^+^, [Cu(POP)(**2**)]^+^, [Cu(xantphos)(**1**)]^+^ and [Cu(xantphos)(**2**)]^+^ cations. Significantly, the 6-substituent is positioned over one phenyl ring of the POP-backbone (Figure 3a,b) or over one arene ring of the xanthene unit (Figure 3c,d), in a similar orientation to that observed for the triazole-containing chain in the solid-state structure of [Cu(xantphos)(**3**)][PF_6_] (Figure 2).

### 2.3. NMR Spectroscopic Properties and Dynamic Behaviour

**Figure 4:**
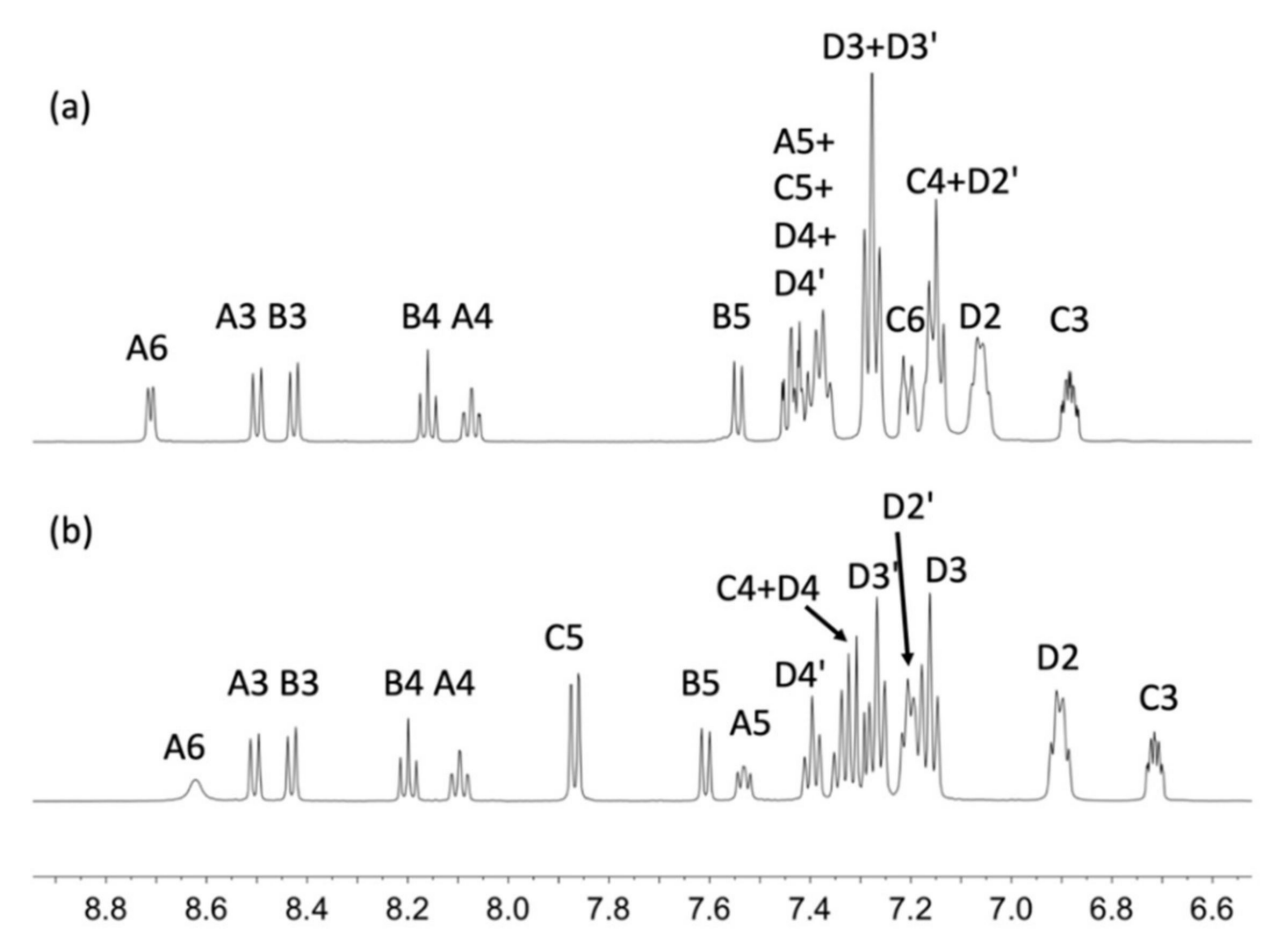
Aromatic regions of the ^1^H-NMR spectra (500 MHz, acetone-d_6_, 298 K) of (**a**) [Cu(POP)(**1**)][PF_6_] and (**b**) [Cu(xantphos)(**1**)][PF_6_].

**Figure 5:**
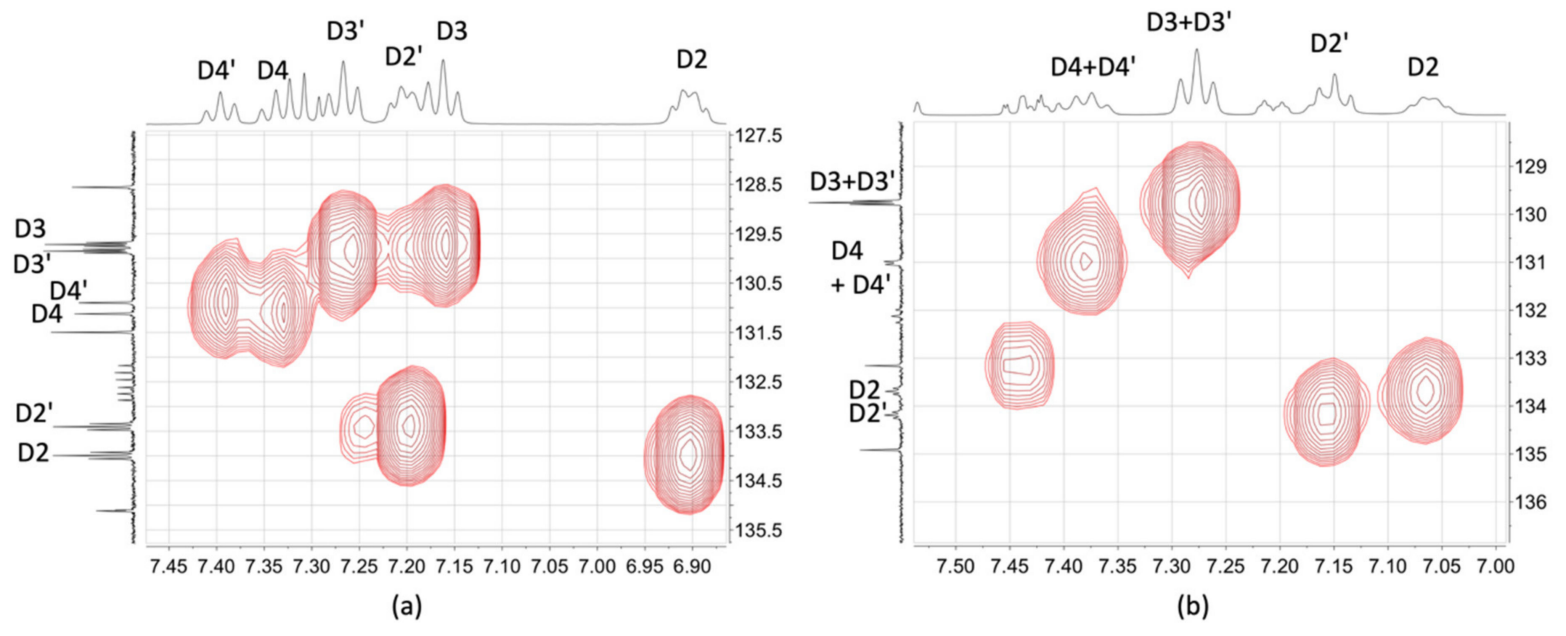
Part of the HMQC spectra (^1^H 500 MHz, ^13^C 126 MHz, acetone-d_6_, 298 K) of (**a**) [Cu(xantphos)(**1**)][PF_6_] and (**b**) [Cu(POP)(**1**)][PF_6_].

The ^1^H- and ^13^C{^1^H}-NMR spectra of the complexes were recorded in acetone-d_6_ and assigned using a combination of COSY, NOESY, HMQC and HMBC methods. ^1^H-NMR, HMQC and HMBC spectra are depicted in Figures S7–S21 in the Supplementary Materials, and full assignments are given in the Material and Methods section. Figure 4 compares the aromatic regions of the ^1^H-NMR spectra of [Cu(POP)(**1**)][PF_6_] and [Cu(xantphos)(**1**)][PF_6_]. Several features are of note. First, on going from Figure 4a,b, the signal for H^C6^ is lost as the CMe_2_ unit is introduced into the P^P ligand backbone (Scheme 1). Secondly, in the spectrum of [Cu(xantphos)(**1**)][PF_6_], the signal for bpy H^A6^ is broadened, and presumably this is associated with the proximity of this proton to two phenyl rings of the PPh_2_ groups of the xantphos ligand (see Figure S6). The third notable distinction between the ^1^H spectra is the splitting of the signals for the phenyl rings into two sets (labelled D and D’), a phenomenon more pronounced in [Cu(xantphos)(**1**)][PF_6_] than [Cu(POP)(**1**)][PF_6_]. The difference is also apparent in the HMQC spectra, expansions of which are displayed in Figure 5.

**Scheme 4:**
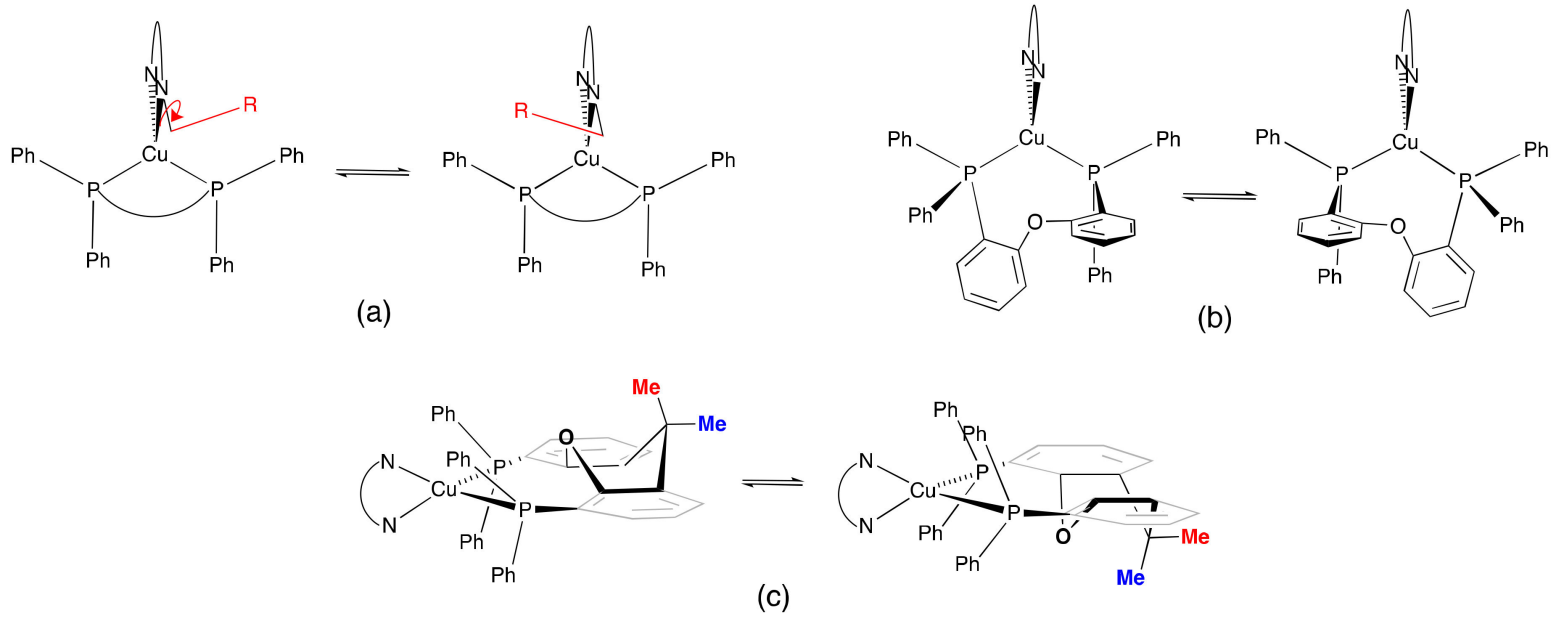
Some of the possible dynamic processes in [Cu(P^P)(N^N)]^+^ cations: (**a**) desymmetrization of the P^P ligand by conformational change of a 6-substituent when N^N = bpy, (**b**) conformational change of the POP backbone, and (**c**) inversion of the xanthene unit in xantphos which exchanges the positions of the methyl groups with respect to the O atom in the xanthene unit.

**Figure 6:**
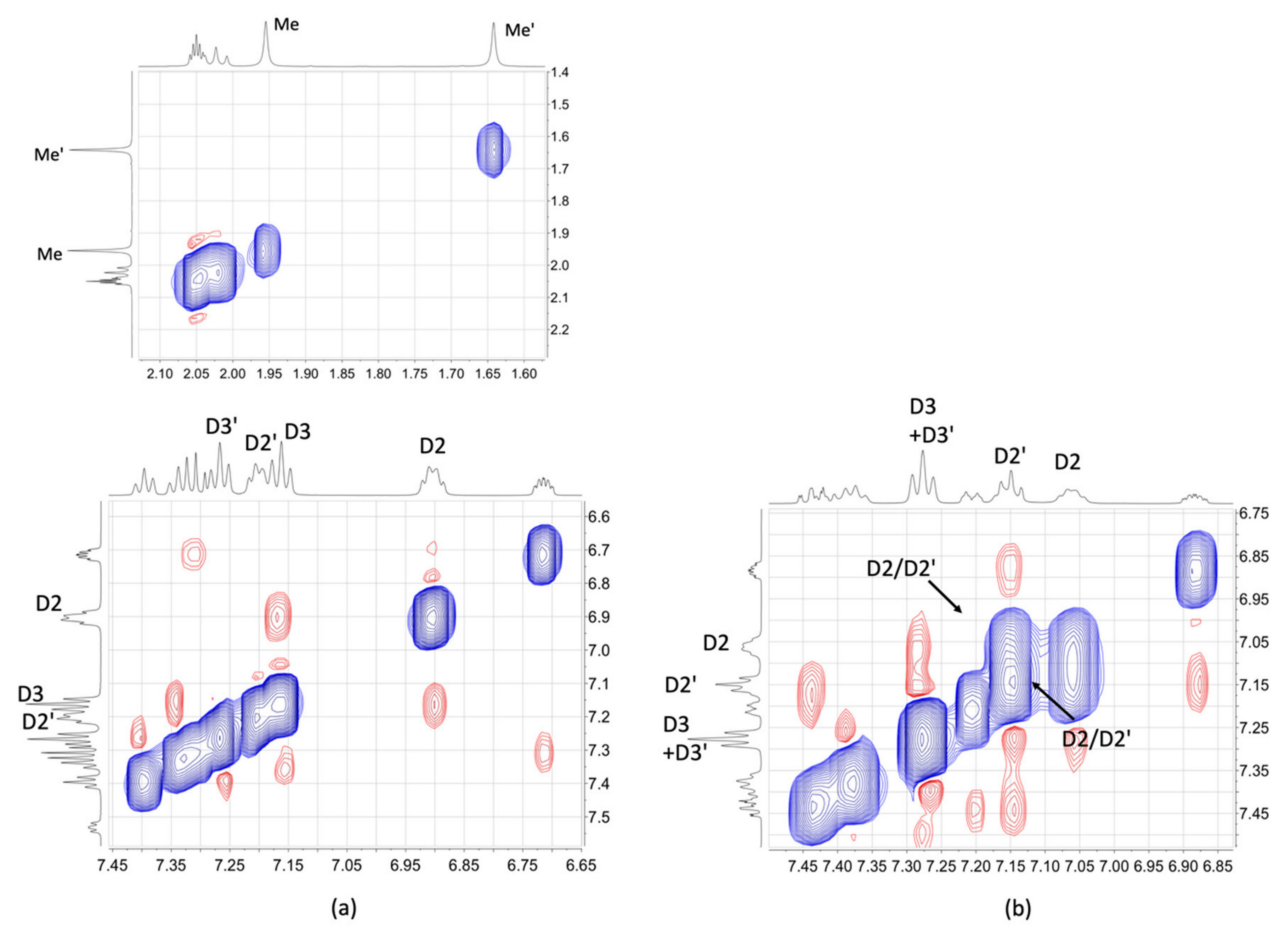
Part of the NOESY spectra (500 MHz, acetone-d_6_, 298 K) of (**a**) [Cu(xantphos)(**1**)][PF_6_] with the methyl region shown at the top, and (**b**) [Cu(POP)(**1**)][PF_6_]. EXSY peaks appear in the opposite phase (blue) to NOESY crosspeaks (red), and are observed between H^D2^ and H^D2′^ in [Cu(POP)(**1**)][PF_6_] but not in [Cu(xantphos)(**1**)][PF_6_].

**Figure 7:**
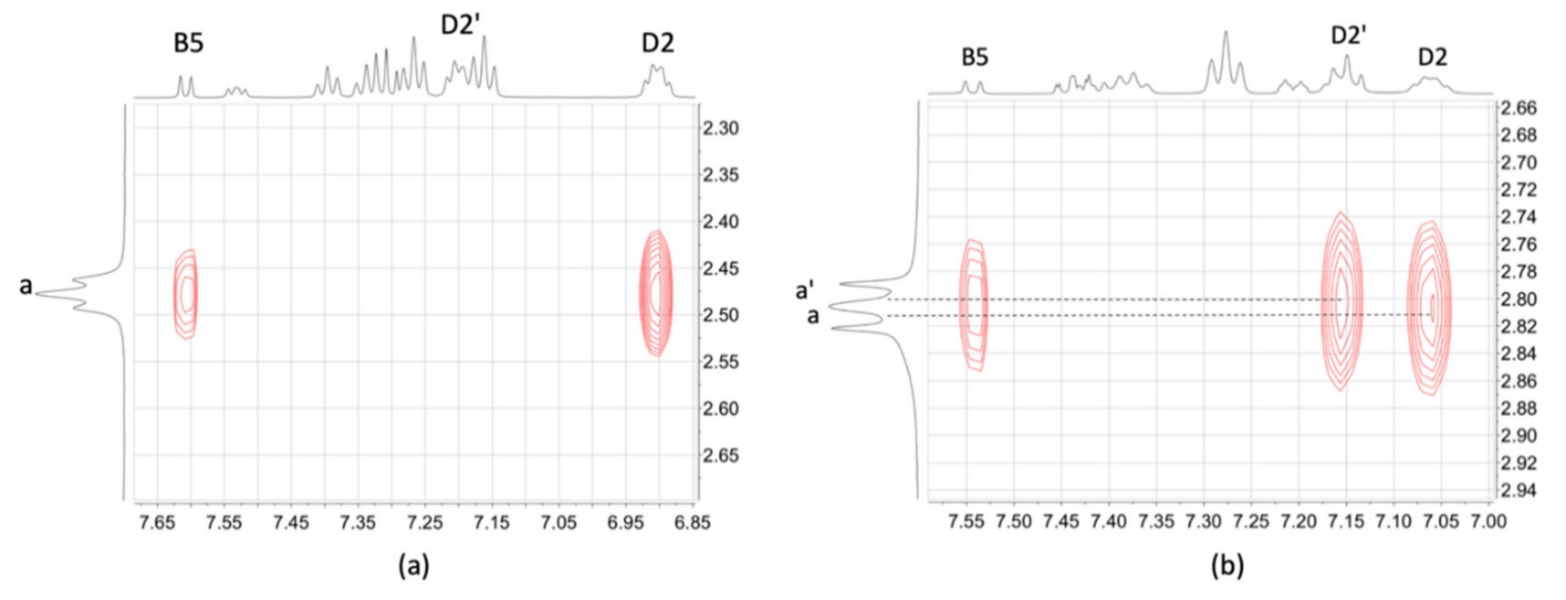
Part of the NOESY spectra (500 MHz, acetone-d_6_, 298 K) of (**a**) [Cu(xantphos)(**1**)][PF_6_] showing NOESY crosspeaks between H^a^ and H^B5^, and H^a^ and H^D2^, and (**b**) [Cu(POP)(**1**)][PF_6_] showing crosspeaks between the diastereotopic protons H^a^/H^a’^ and H^B5^, between H^a^ and H^D2^, and between H^a’^ and H^D2′^.

Non-dissociative dynamic processes to be considered include: (i) rotation of the phenyl rings around P–C bonds which has a very low energy barrier; (ii) conformational change of the 6-substituent from the left- to the right-side of the complex as defined in Scheme 4a; (iii) the conformational change of the POP backbone (Scheme 4b), or conformational change of the xanthene unit in xantphos (Scheme 4c). We may assume that process (i) occurs in all the complexes at 298 K. Processes (ii) and (iii) may be coupled or independent, and the series of complex cations described here reveals different scenarios. First, we compare [Cu(xantphos)(**1**)][PF_6_] and [Cu(POP)(**1**)][PF_6_]. The NOESY spectra in Figure 6 and Figure 7 are particularly informative. The backbone of the xanthene unit is less flexible than that of POP, and the conformational change for xanthene (Scheme 4c) is expected to be a higher energy process than that of POP (Scheme 4b). There are no exchange (EXSY) peaks between the signals for the xantphos H^Me^ and H^Me’^ protons in [Cu(xantphos)(**1**)][PF_6_] (top part of Figure 6a), consistent with no conformational change of the xanthene unit. Additionally, no EXSY peaks are observed between the PPh_2_ H^D2^ and H^D2′^ protons. As Figure 7a shows for [Cu(xantphos)(**1**)][PF_6_], in addition to a NOESY crosspeak to H^B5^(bpy), the protons in the CH_2_ group attached to the bpy unit (H^a^ in Scheme 1) show a NOESY crosspeak with H^D2^, but not with H^D2′^. The data are consistent with the 6-substituent undergoing the conformational change shown in Scheme 4a. This in not coupled with inversion of the xanthene unit (i.e., the process in Scheme 4c is not fast on the NMR timescale at 298 K). In contrast, in the NOESY spectrum of [Cu(POP)(**1**)][PF_6_] (Figure 6b), EXSY peaks are observed between H^D2^ and H^D2′^, indicating that phenyl rings D and D’ exchange, but the process in slow enough on the NMR timescale at 298 K that signals for phenyl groups in two chemically different environments remain distinct. Significantly, the signal for the CH_2_ group attached to bpy in [Cu(POP)(**1**)][PF_6_] is an overlapping doublet of doublets (Figure 7b), indicating that these protons are diastereotopic. Figure 7b shows part of the NOESY spectrum of [Cu(POP)(**1**)][PF_6_], where separate crosspeaks between H^a^ and H^D2^, and between H^a’^ and H^D2′^, in addition to the H^a^/H^a’^ to H^B5^(bpy) crosspeaks are observed. The data for [Cu(POP)(**1**)][PF_6_] are therefore in accord with a combination of the processes shown in (Scheme 4a,b. Analyses of the NMR spectra of [Cu(xantphos)(**2**)][PF_6_] and [Cu(POP)(**2**)][PF_6_] demonstrate that their dynamic behaviours mimic those of [Cu(xantphos)(**1**)][PF_6_] and [Cu(POP)(**1**)][PF_6_], respectively (Figures S22 and S23 in the Supplementary Materials).

**Figure 8:**
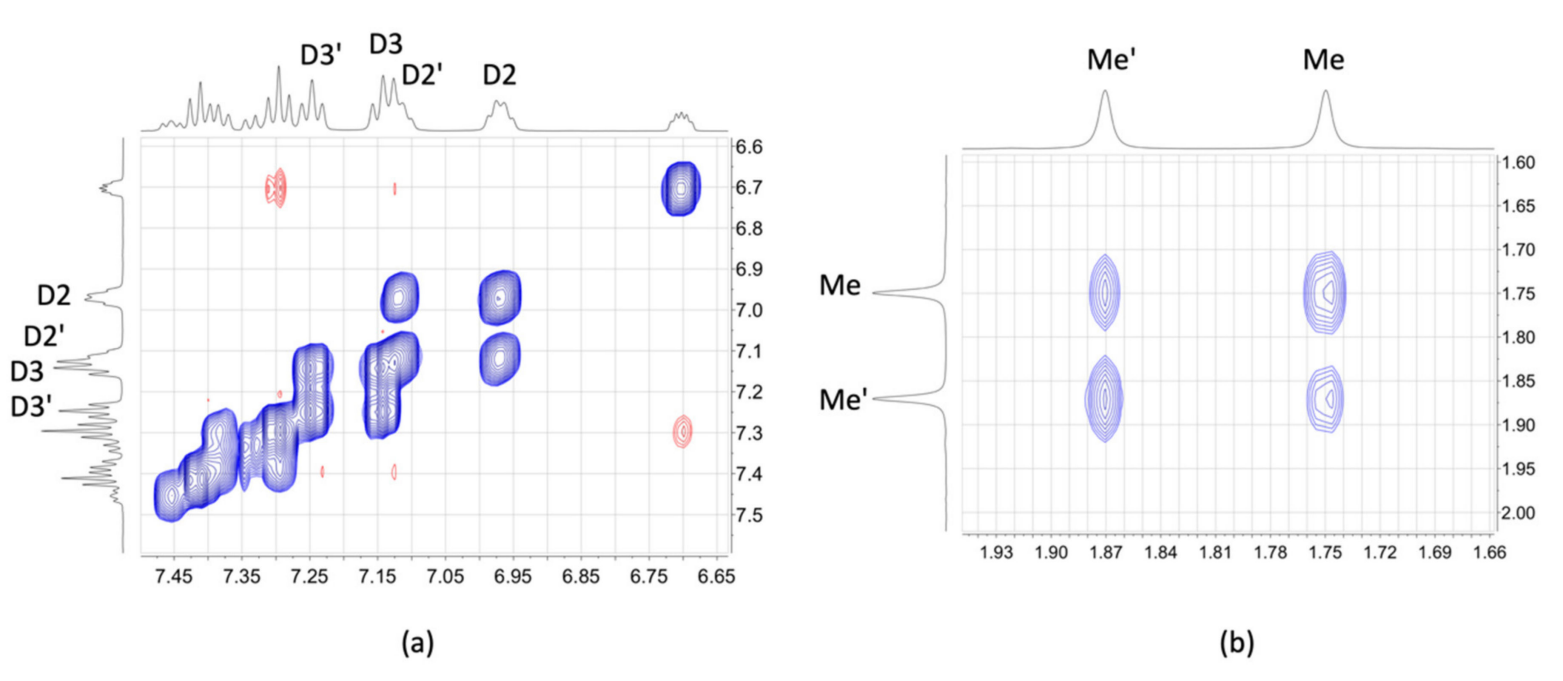
Part of the NOESY spectra (500 MHz, acetone-d_6_, 298 K) of [Cu(xantphos)(**3**)][PF_6_]. (**a**) The EXSY between H^D2^ and H^D2′^, and between H^D3^ and H^D3′^; the EXSY peaks between H^D4^ and H^D4′^ are also visible close to the diagonal. (**b**) EXSY peaks between H^Me^ and H^Me’^ in the xanthene unit.

In contrast to the dynamic behaviour in [Cu(xantphos)(**1**)][PF_6_] and [Cu(xantphos)(**2**)][PF_6_], [Cu(xantphos)(**3**)][PF_6_] undergoes a combination of the processes illustrated in (Scheme 4a,c). The ^1^H- and ^13^C{^1^H}-NMR spectra of an acetone-d_6_ solution of [Cu(xantphos)(**3**)][PF_6_] (298 K) display two sets of signals for the PPh_2_ phenyl rings, and in the NOESY spectrum, EXSY peaks are observed between the H^D2^/H^D2′^, H^D3^/H^D3′^, and H^D4^/H^D4′^ pairs (Figure 8a). Critically, there are EXSY peaks between the two methyl groups of the CMe_2_ unit of xantphos (Figure 8b) confirming that the xanthene unit undergoes a conformation change (Scheme 4c) which is slow on the NMR timescale at 298 K. The NMR spectroscopic data are consistent with this being combined with the ‘flip’ of the 6-substituent (Scheme 4a). It is possible that this is associated with the presence of the aromatic triazole unit in the middle of the substituent chain (see structural discussion and Figure 2).

### 2.4. Electrochemical and Photophysical Properties

The electrochemical behaviour of the copper(I) compounds was investigated using cyclic voltammetry and Table 2 summarizes the observed processes. For comparison, the parent compounds [Cu(POP)(bpy)][PF_6_] and [Cu(xantphos)(bpy)][PF_6_] undergo a reversible copper(I) oxidation at +0.72 V and +0.76 V, respectively [23], and, on going from [Cu(xantphos)(bpy)][PF_6_] to [Cu(xantphos)(6-Mebpy)][PF_6_] to [Cu(xantphos)(6,6′-Me_2_bpy)][PF_6_], oxidation of Cu^+^ occurs at higher potentials (*E*_1/2_^ox^ = +0.76 V, +0.85 V and +0.96 V, respectively) [23]. Although the copper oxidation for each of the compounds reported in Table 2 is irreversible, the *E*_pc_ values are similar to the *E*_1/2_^ox^ for [Cu(xantphos)(6-Mebpy)][PF_6_] to [Cu(xantphos)(6,6′-Me_2_bpy)][PF_6_], and are consistent with the steric demands of the 6-substituents hindering the flattening of the copper coordination sphere which accompanies oxidation from Cu^+^ to Cu^2+^. Each compound also undergoes a reversible bpy-centred reduction, and Figure S24 in the Supplementary Materials shows cyclic voltammograms (CVs) for [Cu(POP)(**2**)][PF_6_] and [Cu(xantphos)(**2**)][PF_6_] as representative data.

**Figure 9:**
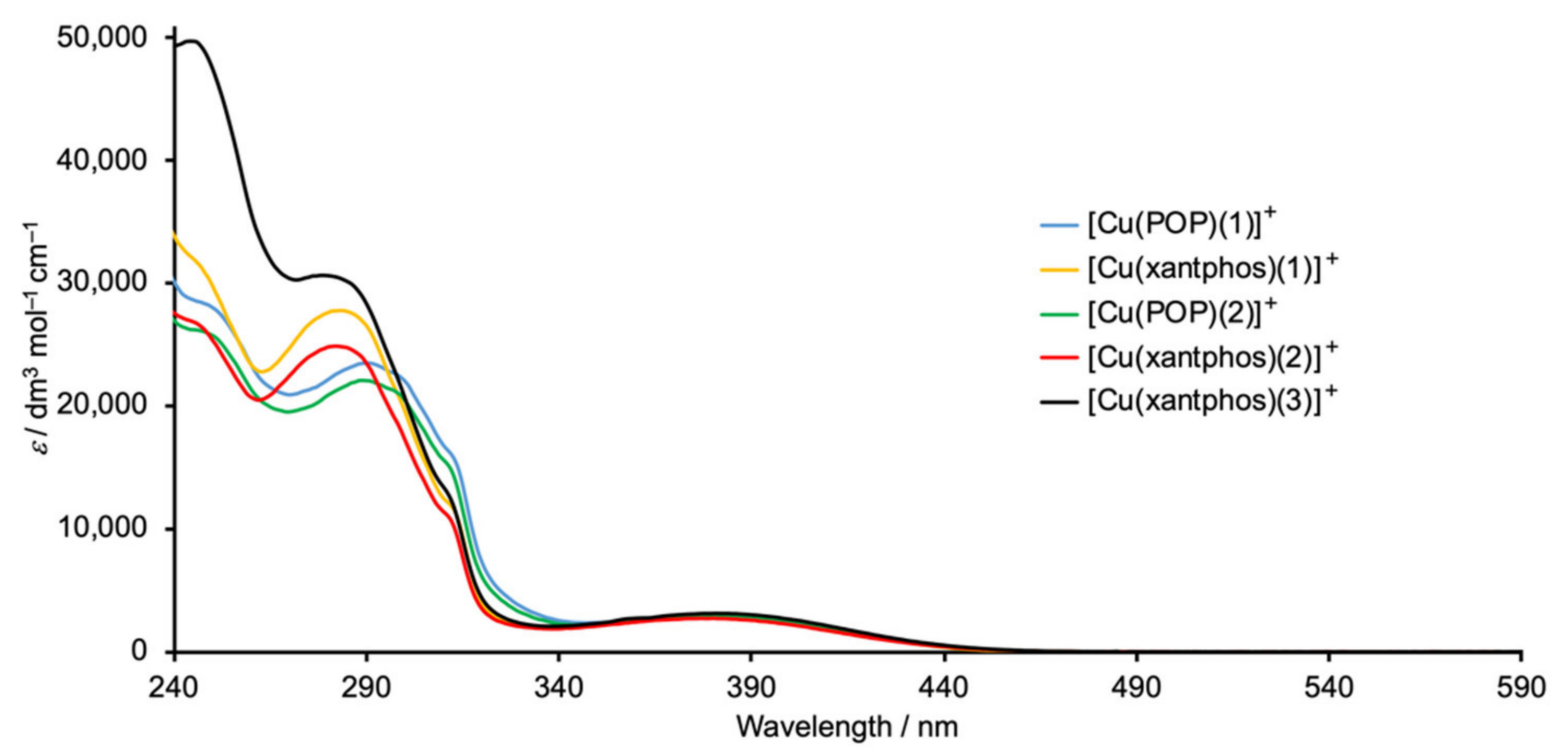
Solution absorption spectra (CH_2_Cl_2_, 2.5 × 10^−5^ mol dm^−3^) of the heteroleptic copper(I) complexes.

The solution absorption spectra of the heteroleptic compounds are displayed in Figure 9 and absorption maxima are given in Table 3. Absorptions below ca. 330 nm arise from ligand-centred transitions. These regions of the spectra of the two POP-containing compounds are similar, and those of the xantphos-containing complexes are also comparable, with the more intense high-energy bands for [Cu(xantphos)(**3**)][PF_6_] being consistent with the presence of the triazole unit. Each of the five complexes exhibits a low-intensity, broad absorption with *λ*_max_ in the range 381–384 nm which is assigned to metal-to-ligand charge-transfer (MLCT).

**Figure 10:**
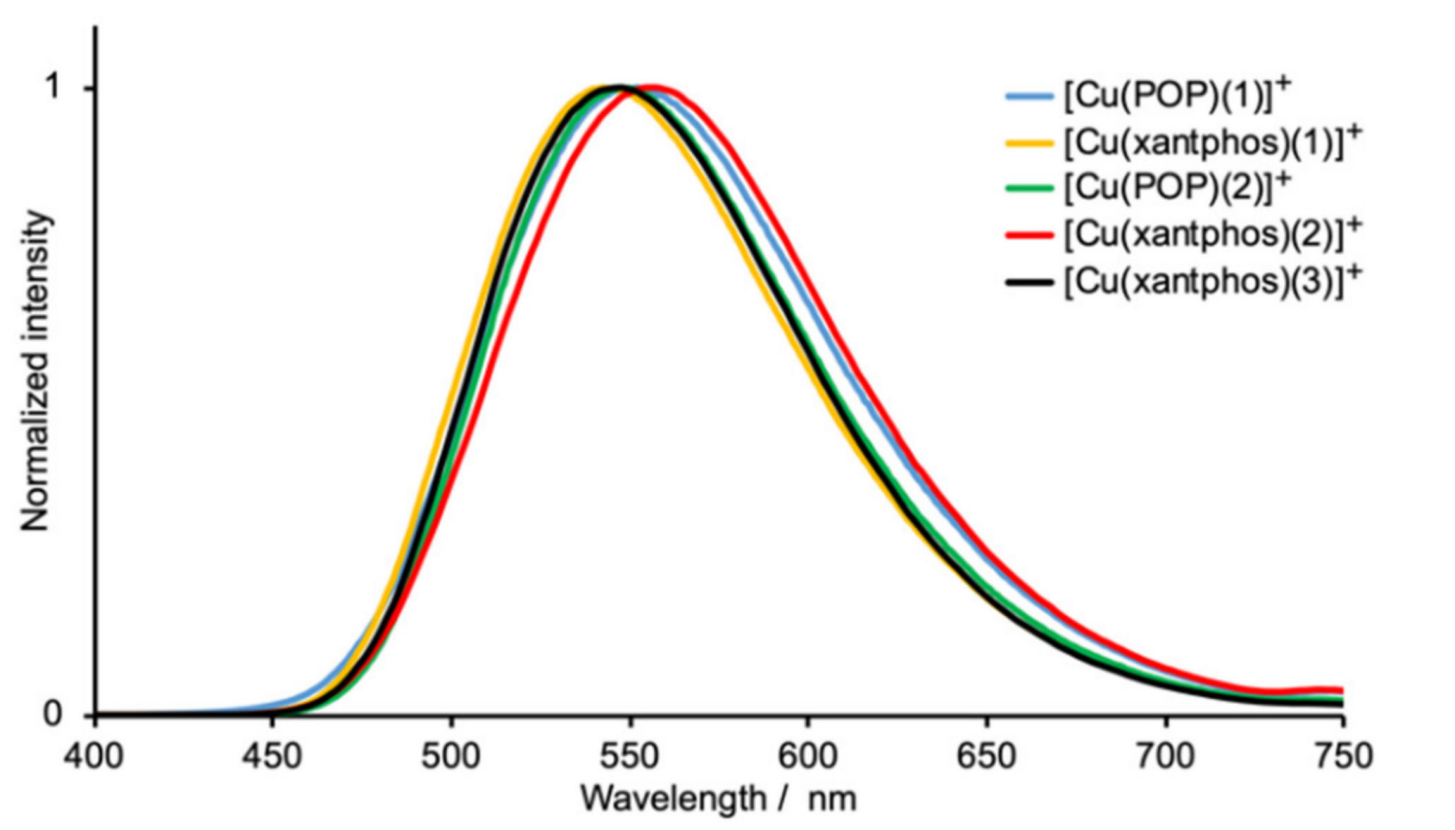
Normalized emission spectra of powdered samples of the heteroleptic copper(I) complexes (*λ*_exc_ = 365 nm).

When de-aerated solutions of the compounds are excited into the MLCT band (*λ*_exc_ = 365 nm), they emit very weakly in the orange region. All solution PLQYs were <1%. As has been described for related heteroleptic [Cu(P^P)(N^N)]^+^ complexes [18], the emissions are assigned to dπ(Cu)→π*(bpy) (^3^MLCT) transitions. The emissions gain in intensity upon going from solution to powdered samples and we focus only on the solid-state data. The emission bands are unstructured (Figure 10) and values of *λ*_max_^em^ are given in Table 4. PLQY values range from 13% for [Cu(POP)(**2**)][PF_6_] to 28% for [Cu(xantphos)(**1**)][PF_6_]. As Table 4 shows, the xantphos-containing compounds have higher PLQY values than those in which P^P is POP. A biexponential fit was used for the lifetime decays (see Table 4), and values of *τ* were all of the same order of magnitude (5.1–8.7 μs). Pleasingly, the PLQYs for [Cu(xantphos)(**1**)][PF_6_], [Cu(xantphos)(**2**)][PF_6_] and [Cu(xantphos)(**2**)][PF_6_] are not significantly lower than that of solid [Cu(xantphos)(6-Mebpy)][PF_6_] (33.8%), and the decay lifetimes (Table 4) are similar to the 9.6 μs reported for [Cu(xantphos)(6-Mebpy)][PF_6_] [18], indicating that the switch from a 6-methyl to longer chain substituent is not detrimental to the photophysical properties.

## 3. Materials and Methods

### 3.1. General

^1^H-, ^13^C{^1^H}- and ^31^P{^1^H}-NMR spectra were recorded using a Bruker Avance III-500 NMR spectrometer (Bruker BioSpin AG, Fällanden, Switzerland) at 298 K. ^1^H and ^13^C NMR chemical shifts were referenced to residual solvent peaks with respect to δ (TMS) = 0 ppm and ^31^P NMR chemical shifts with respect to δ (85% aqueous H_3_PO_4_) = 0 ppm. Shimadzu LCMS-2020 (Shimadzu Schweiz GmbH, 4153 Reinach, Switzerland) and Bruker esquire 3000plus instruments (Bruker BioSpin AG) were used to record electrospray ionization (ESI) mass spectra with samples introduced. Solution absorption and emission spectra were recorded using an Agilent 8453 spectrophotometer (Agilent Technologies Inc., Santa Clara, CA, USA) and Shimadzu RF-5301PC spectrofluorometer (Shimadzu Schweiz GmbH), respectively. PLQYs were measured using a Hamamatsu absolute photoluminescence quantum yield spectrometer C11347 Quantaurus-QY, and emission lifetimes and powder emission spectra were measured using a Compact Fluorescence lifetime Spectrometer C11367 Quantaurus-Tau (Hamamatsu, 4500 Solothurn, Switzerland) with an LED light source (λ_exc_ = 365 nm).

Cyclic voltammograms were recorded using a CH Instruments 900B potentiostat (CH Instruments, city, TX, USA) or a VersaSTAT 3F (AMETEK Princeton Applied Research, Oak Ridge, TN, USA) respectively with [^n^Bu_4_N][PF_6_] (0.1 mol dm^–3^) as the supporting electrolyte. The scan rate was 0.1 V s^–1^, and samples were dissolved in propylene carbonate (ca. 1 × 10^−4^ mol dm^–3^). The working electrode was glassy carbon (ALS Co. Ltd., 131-0033 Tokyo, Japan), the reference electrode was a leakless Ag^+^/AgCl (eDAQ ET069-1) and the counter-electrode was a platinum wire (Advent Research Materials Ltd., OX29 4JA Eynsham, UK). Final potentials were internally referenced with respect to the Fc/Fc^+^ couple.

POP, Cu_2_O, 2-pyridylzinc bromide, *^n^*BuLi, ^i^Pr_2_NH and CuSO_4_**^.^**5H_2_O were purchased from Acros Organics (Chemie Brunschwig AG, Basel, Switzerland). HPF_6_, xantphos, 1-bromo-4-phenylbutane were purchased from Fluorochem (Chemie Brunschwig AG, Basel, Switzerland). (Ph_3_P)_4_Pd, phenylethyne and NaN_3_ were purchased from Sigma Aldrich (Sigma Aldrich Chemie GmbH, Steinheim, Germany).

(2,2′-Bipyridin-6-yl)hexanoic acid (**1**) [20], 6-methyl-2,2′-bipyridine [24], 6-(2-bromoethyl)-2,2′-bipyridine [25] and [Cu(NCMe)_4_][PF_6_] [26] were prepared according to literature procedures.

### 3.2. Compound ***2***

The reaction was carried out in flame-dried glassware on a Schlenk line under an N_2_ atmosphere. Diisopropylamine (152 mg, 0.212 mL, 1.50 mmol, 1.0 eq.) was dissolved in dry THF (5 mL) and the mixture was cooled to −78 °C. *n*-Butyllithium in hexanes (408 mg, 0.600 mL, 2.5 M, 1.50 mmol, 1.0 eq.) was added and the reaction mixture was stirred for 1 h. A solution of 6-methyl-2,2′-bipyridine (93.2 mg, 0.25 mmol, 1.0 eq.) in dry THF (10 mL) was added and the mixture turned dark blue. After the reaction mixture was stirred for another 3 h, a solution of 1-bromo-4-phenylbutane in dry THF (10 mL) was added and the mixture was allowed to warm up to room temperature overnight keeping the vessel submerged in the cooling bath. Then, saturated aqueous NH_4_Cl (15 mL) was added and the mixture was extracted with CH_2_Cl_2_ (3 × 35 mL). The combined organic fractions were dried over MgSO_4_ and filtered. After the solvent was removed under reduced pressure, the residue was purified by flash column chromatography using basic alumina to give 6-(5-phenylpentyl)-2,2′-bipyridine (**2**) (138 mg, 0.46 mmol, 30%) as a colourless oil. For the chromatography, three columns were run: first column: cyclohexane/ethyl acetate, gradient from 3 to 20% ethyl acetate in cyclohexane over 16 column volumes, basic alumina; second column: cyclohexane/toluene (1:1), basic alumina; third column: cyclohexane/ethyl acetate (10:1), basic alumina. ^1^H-NMR (500 MHz, CDCl_3_) *δ*/ppm: 8.66 (m, 1H, H^A6^), 8.51 (d, *J* = 8.0 Hz, 1H, H^A3^), 8.29 (d, *J* = 7.9 Hz, 1H, H^B3^), 7.90 (dd, *J* = 9.4, 7.7 Hz, 1H, H^A4^), 7.80 (dd, *J* = 7.7, 7.7 Hz, 1H, H^B4^), 7.39 (m, 1H, H^A5^), 7.27 (m, 1H, H^B5^), 7.23 (m, 2H, H^F3^), 7.20 (m, 2H, H^F2^), 7.14 (m, 1H, H^F4^), 2.86 (t, *J* = 7.6, Hz, 2H, H^a^), 2.62 (t, 7.6, Hz, 2H, H^e^), 1.86 (m, 2H, H^b^), 1.70 (m, 2H, H^d^), 1.45 (m, 2H, H^c^). ^13^C{^1^H}-NMR (126 MHz, CDCl_3_) *δ*/ppm: 157.2 (C^A2^), 156.2 (C^B2^), 150.0 (C^A6^), 143.5 (C^E1^), 138.0 (C^B4^), 137.6 (C^A4^), 129.2 (C^E2^), 129.1 (C^E3^), 126.5 (C^B6^), 126.4 (C^E4^), 124.5 (C^A5^), 123.7 (C^B5^), 121.4 (C^A3^), 118.6 (C^B3^), 38.7 (C^a^), 36.4 (C^e^), 32.6 (C^d^), 30.2 (C^b^), 29.6 (C^c^). ESI MS: *m*/*z* 303.10 [M + H]^+^ (base peak, calc. 303.19), 325.11 [M + Na]^+^ (calc. 325.17).

### 3.3. Compound ***3***

6-(2-Bromoethyl)-2,2′-bipyridine (130 mg, 0.49 mmol, 1.0 eq.) was dissolved in DMF (2 mL), and NaN_3_ (48.2 mg, 0.74 mmol, 1.5 eq.) was added. The reaction mixture was stirred for ca. 15 h, and then water (5 mL) was added and the mixture was extracted with CH_2_Cl_2_ (4 × 15 mL). The solvent volume was reduced under vacuum. Phenylethyne (101 mg, 0.99 mmol, 2.0 eq.), water (1 mL) and *t*-BuOH (5mL) were added, followed by CuSO_4_**^.^**5H_2_O (12.3 mg, 0.049 mmol, 0.1 eq.) and sodium ascorbate (97.9 mg, 0.49 mmol, 1.0 eq.). The mixture was stirred at 35 °C for 72 h. The ^1^H NMR spectrum of the crude material indicated an incomplete conversion, and therefore more CuSO_4_**^.^**5H_2_O (123 mg, 0.49 mmol, 1.0 eq.) and sodium ascorbate (97.9 mg, 0.49 mmol, 1.0 eq.) were added. The reaction mixture was stirred at 35 °C for 5 h. Water (10 mL) was added and the mixture was extracted with CH_2_Cl_2_ (3 × 20 mL). The combined organic layers were washed with water (2 × 30 mL) and dried over MgSO_4_. The solvent was removed under vacuum and the residue was dissolved in CH_2_Cl_2_ (10 mL). The organic layer was washed with a mixture (2 × 20 mL) of aqueous H_2_O_2_ (ca 1 %) and Na_4_EDTA (ca. 1% in 2 M aqueous NaOH). The organic layer was dried over MgSO_4_ and the solvent was removed under vacuum. The product was purified by column chromatography (Alox 90 basic, cyclohexane: ethyl acetate 2: 1) to give **3** as a white solid (87.0 mg, 0.27 mmol, 54%). ^1^H-NMR (500 MHz, CDCl_3_) *δ*/ppm: 8.69 (d, *J* = 4.1 Hz, 1H, H^A6^), 8.41 (d, *J* = 7.9 Hz, 1H, H^A3^), 8.28 (d, *J* = 7.9 Hz, 1H, H^B3^), 7.79 (t, *J* = 7.7 Hz, 1H, H^A4^), 7.74 (d, *J* = 7.9 Hz, 2H, H^F2^), 7.71 (t, *J* = 7.8 Hz, 1H, H^B4^), 7.62 (s, 1H, H^E5^), 7.37 (t, *J* = 7.6 Hz, 2H, H^F3^), 7.34−7.27 (overlapping m, 2H, H^A5+F4^), 7.09 (d, *J* = 7.6 Hz, 1H, H^B5^), 4.98 (t, *J* = 6.9 Hz, 2H, H^b^), 3.50 (t, *J* = 6.9 Hz, 2H, H^a^). ^13^C{^1^H}-NMR (126 MHz, CDCl_3_) *δ*/ppm: 156.5 (C^B6^), 156.1 (C^A2^), 156.0 (C^B2^), 149.4 (C^A6^), 147.6 (C^E4^), 137.8 (C^B4^), 137.0 (C^A4^), 130.8 (C^F1^), 128.9 (C^F3^), 128.1 (C^F4^), 125.8 (C^F2^), 124.0 (C^A5^), 123.8 (C^B5^), 121.2 (C^A3^), 120.2 (C^E5^), 119.5 (C^B3^), 49.5 (C^b^), 38.4 (C^a^). ESI MS: *m*/*z* 328.08 [M + H]^+^ (base peak calc. 328.16), 350.08 [M + Na]^+^ (calc. 350.14).

### 3.4. [Cu(POP)(**1**)][PF_6_]

[Cu(MeCN)_4_][PF_6_] (93.2 mg, 0.25 mmol, 1.0 eq.) and POP (148 mg, 0.28 mmol, 1.1 eq.) were dissolved in CH_2_Cl_2_ (30 mL) and the mixture stirred for 1 h. Compound **1** (67.6 mg, 0.25 mmol, 1.0 eq.) was added and the mixture turned red, then orange while it was stirred for 1 h. The colour indicated the presence of homoleptic [Cu(**1**)_2_][PF_6_] and therefore additional POP (26.9 mg, 0.05 mmol, 0.2 eq.) was added. The mixture turned yellow and after stirring for 1.5 h, the mixture was filtered and the volume was reduced in vacuo. The product was precipitated by addition of Et_2_O and the solid was washed by sonication in Et_2_O (4 × 15 mL). After recrystallization, [Cu(POP)(**1**)][PF_6_] was isolated as a yellow solid (213 mg, 0.21 mmol, 84%). ^1^H-NMR (500 MHz, acetone-d_6_) *δ*/ppm: 10.45 (br s, 1H, H^COOH^), 8.71 (d, *J* = 5.0 Hz, 1H, H^A6^), 8.50 (d, *J* = 8.1 Hz, 1H, H^A3^), 8.43 (d, *J* = 7.9 Hz, 1H, H^B3^), 8.16 (t, *J* = 7.9 Hz, 1H, H^B4^), 8.07 (td, *J* = 7.8, 1.6 Hz, 1H, H^A4^), 7.54 (d, *J* = 7.9 Hz, 1H, H^B5^), 7.46–7.35 (overlapping m, 7H, H^A5+C5+D4+D4′^), 7.28 (t, *J* = 7.6 Hz, 8H, H^D3+D3′^), 7.21 (m, 2H, H^C6^), 7.18–7.13 (overlapping m, 6H, H^C4+D2′^), 7.07 (m, 4H, H^D2^), 6.88 (dtd, *J* = 7.8, 4.0, 1.7 Hz, 2H, H^C3^), 2.81 (dd, *J* = 8.2, 8.2 Hz, 2H, H^a^), 2.12 (t, *J* = 7.4 Hz, 2H, H^e^), 1.31 (m, 2H, H^d^), 1.25 (m, 2H, H^b^), 0.85 (m, 3H, H^c^). ^13^C{^1^H}-NMR (126 MHz, acetone-*d_6_*) *δ*/ppm: 174.5 (C^COOH^), 163.7 (C^B6^), 158.8 (t, *J*_PC_ = 6 Hz, C^C1^), 153.6 (t, *J*_PC_ = 2 Hz, C^A2^), 152.4 (C^B2^), 150.3 (C^A6^), 140.1 (C^B4^), 139.7 (C^A4^), 134.9 (C^C3^), 134.2 (t, *J*_PC_ = 8 Hz, C^D2′^), 133.7 (t, *J*_PC_ = 8 Hz, C^D2^), 133.2 (C^C5^), 132.1 (t, *J*_PC_ = 17 Hz, C^D1^), 131.1 (C^D4′^), 131.0 (C^D4^), 129.8 (t, *J*_PC_ = 5 Hz, C^D3+D3′^), 126.7 (C^A5^), 126.1 (t, *J*_PC_ = 2 Hz, C^C2^), 125.3 (C^B5^), 125.1 (t, *J*_PC_ = 14 Hz, C^C2^), 123.6 (C^A3^), 121.4 (t, *J*_PC_ = 2 Hz, C^C6^), 121.2 (C^B3^), 40.8 (C^a^), 34.0 (C^e^), 29.5 (C^c^), 28.7 (C^b^), 25.5 (C^d^). ^31^P{^1^H}-NMR (202 Hz, acetone-*d_6_*) *δ*/ppm: −13.1, −144.3 (septet, *J*_PF_ = 707 Hz, [PF_6_]^−^). ESI MS: *m*/*z* 871.21 [M−PF_6_]^+^ (base peak, calc. 871.23). UV-Vis (CH_2_Cl_2_, 2.5 × 10^–5^ mol dm^–3^): *λ*/nm (*ε*/dm^3^ mol^–1^ cm^–1^) 250 (28,000), 292 (23,500), 380 (3000). Found: C 61.28, H 4.71, N 2.66; C_52_H_46_CuF_6_N_2_O_3_P_3_ requires C 61.39, H 4.56, N 2.75%.

### 3.5. [Cu(xantphos)(**1**)][PF_6_]

[Cu(MeCN)_4_][PF_6_] (93.2 mg, 0.25 mmol, 1.0 eq.) was dissolved in CH_2_Cl_2_ (15 mL). A solution of xantphos (145 mg, 0.25 mmol, 1.0 eq.) and **1** (67.6 mg, 0.25 mmol, 1.0 eq.) in CH_2_Cl_2_ (30 mL) was added and the mixture turned red then yellow while being stirred for 2 h at room temperature. The yellow solution was filtered and the solvent was removed from the filtrate *in vacuo* leaving a yellow solid that was washed with Et_2_O (4 × 10 mL), recrystallized and dried under vacuum. [Cu(xantphos)(**1**)][PF_6_] (259 mg, 0.25 mmol, 98%) was isolated as a yellow solid. ^1^H-NMR (500 MHz, acetone-d_6_) *δ*/ppm: 10.41 (s, 1H, H^COOH^), 8.62 (br, 1H, H^A6^), 8.50 (d, *J* = 8.2 Hz, 1H, H^A3^), 8.43 (d, *J* = 7.9 Hz, 1H, H^B3^), 8.20 (t, *J* = 7.9 Hz, 1H, H^B4^), 8.10 (td, *J* = 7.8, 1.6 Hz, 1H, H^A4^), 7.87 (dd, *J* = 7.8, 1.5 Hz, 2H, H^C5^), 7.61 (d, *J* = 7.8 Hz, 1H, H^B5^), 7.53 (dd, *J* = 7.6, 5.1 Hz, 1H, H^A5^), 7.40 (t, *J* = 7.4 Hz, 2H, H^D4′^), 7.36–7.29 (overlapping m, 4H, H^C4+D4^), 7.27 (m, 4H, H^D3′^), 7.20 (m, 4H, H^D2′^), 7.16 (m, 4H, H^D3^), 6.90 (m, 4H, H^D2^), 6.71 (m, 2H, H^C3^), 2.48 (t, *J* = 7.6 Hz, 2H, H^a^), 2.02 (t, *J* = 7.7 Hz, 2H, H^e^), 1.95 (s, 3H, H^xantphos-Me^), 1.64 (s, 3H, H^xantphos-Me’^), 1.23–1.12 (m, 4H, H^b+c^), 0.51 (m, 2H, H^d^). ^13^C{^1^H}- NMR (126 MHz, acetone-d_6_) *δ*/ppm: 174.3 (C^COOH^), 163.0 (C^B6^), 155.9 (t, *J*_PC_ = 6 Hz, C^C1^), 153.5 (t, *J*_PC_ = 2 Hz, C^A2^), 152.3 (t, *J*_PC_ = 2 Hz, C^B2^), 150.0 (C^A6^), 140.3 (C^B4^), 139.9 (C^A4^), 135.1 (t, *J*_PC_ = 2 Hz, C^C6^), 134.0 (t, *J*_PC_ = 8 Hz, C^D2^), 133.4 (t, *J*_PC_ = 8 Hz, C^D2′^), 132.8 (m, C^D1′^), 132.3 (m, C^D1^), 131.5 (C^C3^), 131.1 (C^D4^), 130.9 (C^D4′^), 129.9 (t, *J*_PC_ = 5 Hz, C^D3′^), 129.7 (t, *J*_PC_ = 5 Hz, C^D3^), 128.6 (C^C5^), 127.0 (C^A5^), 126.3 (t, *J*_PC_ = 2 Hz, C^C4^), 125.5 (C^B5^), 123.8 (C^A3^), 121.50 (t, *J* = 14 Hz, C^C2^), 121.46 (C^B3^), 41.2 (C^a^), 37.0 (C^xantphos-bridge^), 33.8 (br, C^e^), 30.9 (br, C^xantphos-Me’^), 28.8 (C^d^), 28.5 (C^b^), 25.7 (br, C^xantphos-Me^), 25.5 (C^c^). ^31^P{^1^H}-NMR (202 Hz, acetone-*d_6_*) *δ*/ppm: −13.1, −144.2 (septet, *J*_PF_ = 708 Hz, [PF_6_]^−^). ESI MS: *m/z* 911.21 [M − PF_6_]^+^ (base peak, calc. 911.26), 641.10 [Cu(xantphos)]^+^ (calc. 641.12). UV-Vis (CH_2_Cl_2_, 2.5 × 10^–5^ mol dm^–3^): *λ*/nm (*ε*/dm^3^ mol^–1^ cm^–1^) 248sh (31,500), 285 (28,000), 381 (3100). Satisfactory elemental analysis could not be obtained.

### 3.6. [Cu(POP)(**2**)][PF_6_]

[Cu(MeCN)_4_][PF_6_] (86.8 mg, 0.23 mmol, 1.0 eq.) and POP (126 mg, 0.23 mmol, 1.0 eq.) were dissolved in CH_2_Cl_2_ (30 mL) and the mixture was stirred for 2 h. Compound **2** (70.6 mg, 0.23 mmol, 1.0 eq.) was added and the mixture turned red, then yellow while it was stirred for 2 h. The mixture was filtered and the volume was reduced under vacuum. The product was purified by preparative crystallization from CH_2_Cl_2_ by addition of Et_2_O. After drying, [Cu(POP)(**2**)][PF_6_] was isolated as a yellow solid (191 mg, 0.18 mmol, 78%). ^1^H- NMR (500 MHz, acetone-d_6_) *δ*/ppm: 8.68 (d, *J* = 5.1 Hz, 1H, H^A6^), 8.50 (d, *J* = 8.1 Hz, 1H, H^A3^), 8.42 (d, *J* = 7.8 Hz, 1H, H^B3^), 8.15 (dd, *J* = 7.9, 7.9 Hz, 1H, H^B4^), 8.07 (dd, *J* = 7.9, 8.0 Hz, 1H, H^A4^), 7.53 (d, *J* = 7.8 Hz, 1H, H^B5^), 7.45–7.35 (overlapping m, 7H, H^D4+D4′+C5+A5^), 7.26 (m, 10H, H^D3+D3′+E3^), 7.19–7.12 (overlapping m, 11H, H^E2+E4+C4+C6+D2′^), 7.05 (m, 4H, H^D2^), 6.88 (m, 1H, H^C3^), 2.80 (m, 2H, H^a^), 2.44 (m, 2H, H^e^), 1.28 (m, 2H, H^d^), 1.27 (m, 2H, H^b^), 0.85 (m, 2H, H^c^). ^13^C{^1^H}-NMR (126 MHz, acetone-d_6_) *δ*/ppm: 163.8 (C^B6^), 158.8 (t, *J*_PC_ = 6 Hz, C^C1^), 153.6 (C^A2^), 152.4 (C^B2^), 150.2 (C^A6^), 143.3 (C^E1^), 140.1 (C^B4^), 139.7 (C^A4^), 135.0 (C^C3^), 134.2 (t, *J*_PC_ = 8 Hz, C^D2′^), 133.7 (t, *J*_PC_ = 8 Hz, C^D2^), 133.2 (C^C5^), 132.1 (m, C^D1+D1′^), 131.0 (C^D4+D4′^), 129.8 (t, *J*_PC_ = 5 Hz, C^D3+D3′^), 129.3 (C^E2/E3^), 129.1 (C^E2/E3^), 126.6 (C^A5^), 126.5 (C^E4^), 126.1 (t, *J*_PC_ = 2 Hz, C^C4^), 125.4 (C^B5^), 125.0 (t, *J*_PC_ = 14 Hz, C^C2^), 123.6 (C^A3^), 121.3 (t, *J*_PC_ = 2 Hz, C^C6^), 121.2 (C^B3^), 40.9 (C^a^), 36.3 (C^e^), 32.1 (C^d^), 29.5 (C^c^), 29.0 (C^b^). ^31^P{^1^H}-NMR (202 MHz, 298 K, acetone-d_6_) *δ*/ppm: −13.1, −144.2 (septet, *J*_PF_ = 707 Hz, [PF_6_]^–^). ESI-MS *m*/*z*: 903.25 [M − PF_6_]^+^ (base peak, calc. 903.27), 601.09 [Cu(POP)]^+^ (calc. 601.09). UV-Vis (CH_2_Cl_2_, 2.5 × 10^–5^ mol dm^–3^): *λ*/nm (*ε*/dm^3^ mol^–1^ cm^–1^) 250 (25,750), 290 (22,000), 383 (3000). Found: C 64.44, H 4.91, N 2.67; C_57_H_50_CuF_6_N_2_OP_3_ requires C 65.23, H 4.80, N 2.67.

### 3.7. [Cu(xantphos)(**2**)][PF_6_]

[Cu(MeCN)_4_][PF_6_] (86.1 mg, 0.23 mmol, 1.0 eq.), xantphos (134 mg, 0.23 mmol, 1.0 eq.) and **2** (70.0 mg, 0.23 mmol, 1.0 eq.) were dissolved in CH_2_Cl_2_ (30 mL). The mixture turned red, then yellow while it was stirred for 2 h. The mixture was filtered and the volume was reduced under vacuum. The product was purified by preparative crystallization from CH_2_Cl_2_ by addition of Et_2_O. AFter drying, [Cu(xantphos)(**2**)][PF_6_] was isolated as a yellow solid (204 mg, 0.19 mmol, 81%). ^1^H-NMR (500 MHz, acetone-d_6_) *δ*/ppm: 8.59 (m, 1H, H^A6^), 8.51 (d, *J* = 8.2 Hz, 1H, H^A3^), 8.43 (d, *J* = 7.9 Hz, 1H, H^B3^), 8.19 (dd, *J* = 7.9, 7.9 Hz, 1H, H^B4^), 8.10 (dd, *J* = 9.1, 7.7 Hz, 1H, H^A4^), 7.84 (dd, *J* = 7.9, 1.4 Hz, 2H, H^C5^), 7.58 (d, *J* = 7.7 Hz, 1H, H^B5^), 7.52 (dd, *J* = 6.8, 5.5 Hz, 1H, H^A5^), 7.39 (t, *J* = 7.4 Hz, 2H, H^D4′^), 7.34 (t, *J* = 7.5 Hz, 2H, H^D4^), 7.26 (m, 2H, H^C4^), 7.25 (m, 4H, H^D3′^), 7.23 (m, 2H, H^E3^), 7.19 (m, 4H, H^D2′^), 7.15 (m, 4H, H^D3^), 7.15 (m, 1H, H^E4^), 7.05 (m, 2H, H^E2^), 6.91 (m, 4H, H^D2^), 6.7 (m, 1H, H^C3^), 2.49 (dd, *J* = 7.6 Hz, 2H, H^a^), 2.34 (dd, *J* = 7.6 Hz, 2H, H^e^), 1.93 (s, 3H, H^xantphos-Me’^), 1.65 (s, 3H, H^xantphos-Me^), 1.22 (m, 2H, H^d^), 1.19 (m, 2H, H^b^), 0.55 (m, 2H, H^c^). ^13^C{^1^H}-NMR (126 MHz, acetone-d_6_) *δ*/ppm: 153.5 (C^A2^), 163.1 (C^B6^), 155.8 (t, *J*_PC_ = 6 Hz, C^C1^), 152.3 (C^B2^), 150.0 (C^A6^), 143.2 (C^E1^), 140.3 (C^B4^), 139.9 (C^A4^), 135.1 (t, *J*_PC_ = 2 Hz, C^C6^), 134.0 (t, *J*_PC_ = 8 Hz, C^D2^), 133.4 (t, *J*_PC_ = 8 Hz, C^D2′^), 132.8 (t, *J*_PC_ = 16 Hz, C^D1′^), 132.4 (t, *J*_PC_ = 18 Hz, C^D1^), 131.5 (C^C3^), 131.1 (C^D4^), 130.9 (C^D4′^), 129.9 (t, *J*_PC_ = 5 Hz, C^D3′^), 129.7 (t, *J*_PC_ = 5 Hz, C^D3^), 129.7 (C^E3^), 129.1 (C^E2^), 128.6 (C^C5^), 127.0 (C^A5^), 126.5 (C^E4^), 126.3 (t, *J* = 2 Hz, C^C4^), 125.5 (C^B5^), 123.8 (C^A3^), 121.5 (t, *J*_PC_ = 13 Hz, C^C2^), 121.4 (C^B3^), 41.3 (C^a^), 36.9 (C^xantphos-bridge^), 36.1 (C^e^), 32.2 (C^d^), 30.8 (C^xantphos-Me^), 28.9 (C^c^), 28.7 (C^b^), 25.9 (C^xantphos-Me’^). ^31^P{^1^H}-NMR (202 MHz, 298 K, acetone-d_6_) *δ*/ppm: −13.1, −144.2 (septet, *J*_PF_ = 707 Hz). ESI-MS *m*/*z*: 943.32 [M − PF_6_]^+^ (base peak, calc. 943.30), 641.11 [Cu(xantphos)]^+^ (calc. 641.12). UV-Vis (CH_2_Cl_2_, 2.5 × 10^–5^ mol dm^–3^): *λ*/nm (*ε*/dm^3^ mol^–1^ cm^–1^) 248 (26,500), 284 (25,000), 383 (2750). Found: C 65.50, H 4.92, N 2.57; C_60_H_54_CuF_6_N_2_OP_3_ requires C 66.14, H 5.00, N 2.57.

### 3.8. [Cu(xantphos)(**3**)][PF_6_]

[Cu(MeCN)_4_][PF_6_] (42.1 mg, 0.11 mmol, 1.0 eq.) was dissolved in CH_2_Cl_2_ (15 mL). A solution of xantphos (65.4 mg, 0.11 mmol, 1.0 eq.) and **3** (37.0 mg, 0.11 mmol, 1.0 eq.) was added and the mixture turned red then yellow as it was stirred for 2 h at room temperature. The reaction mixture was filtered and the solvent was removed from the filtrate to yield a yellow solid that was washed with Et_2_O (4 × 10 mL), recrystallized and dried under vacuum. [Cu(xantphos)(**3**)][PF_6_] was isolated as a yellow solid (125 mg, 0.11 mmol, 99%). ^1^H-NMR (500 MHz, acetone-d_6_) δ/ppm: 8.56 (d, *J* = 8.2 Hz, 1H, H^A3^), 8.49 (d, *J* = 7.9 Hz, 1H, H^B3^), 8.32 (br s, 1H, H^A6^), 8.14 (t, J = 7.9 Hz, 1H, H^B4^), 8.11 (t, *J* = 7.9 Hz, 1H, H^A4^), 7.87 (d, *J* = 7.8 Hz, 2H, H^C5^), 7.73 (d, *J* = 7.7 Hz, 2H, H^F2^), 7.68 (s, 1H, H^E5^), 7.45 (dd, *J* = 7.6, 5.3 Hz, 1H, H^A5^), 7.43–7.36 (overlapping m, 5H, H^B5+D4+F3^), 7.34−7.27 (overlapping m, 5H, H^F4+C4+D4′^), 7.25 (m, 4H, H^D3′^), 7.18−7.09 (overlapping m, 8H, H^D2′+D3^), 6.97 (m, 4H, H^D2^), 6.70 (m, 2H, H^C3^), 4.20 (t, *J* = 6.9 Hz, 2H, H^b^), 3.31 (br t, 3H, H^a^), 1.87 (s, 3H, H^xantphos-Me^), 1.75 (s, 3H, H^xantphos-Me’^). ^13^C{^1^H}-NMR (126 MHz, acetone-d_6_) δ/ppm: 158.6 (C^B6^), 155.7 (t, *J*_PC_ = 6.3 Hz, C^C1^), 153.3 (C^A2^), 153.0 (C^B2^), 149.8 (C^A6^), 147.9 (C^E4^), 140.5 (C^B4^), 140.0 (C^A4^), 135.1 (t, *J*_PC_ = 2 Hz, C^C6^), 133.9 (t, *J*_PC_ = 8 Hz, C^D2^), 133.5 (t, *J*_PC_ = 8 Hz, C^D2′^), 132.5 (t, *J*_PC_ = 17 Hz, C^D1′^), 132.0 (t, *J*_PC_ = 18 Hz, C^D1^), 131.9 (C^F1^), 131.7 (C^C3^), 131.11 (C^D4′^), 131.06 (C^D4^), 129.92 (t, *J*_PC_ = 5 Hz, C^D3′^), 129.88 (t, *J*_PC_ = 5 Hz, C^D3^), 129.7 (C^F3^), 128.9 (C^F4^), 128.8 (C^C5^), 127.1 (C^A5^), 126.5 (t, *J*_PC_ = 2 Hz, C^C4^), 126.3 (C^F2^), 126.0 (C^B5^), 124.1 (C^A3^), 122.4 (C^B3^), 121.23 (t, *J*_PC_ = 14 Hz, C^C2^), 121.17 (C^E5^), 48.1 (C^b^), 40.9 (C^a^), 37.0 (C^xantphos-bridge^), 29.6 (C^xantphos-Me’^), 27.2 (C^xantphos-Me^). ^31^P{^1^H}-NMR (202 Hz, acetone-d_6_) δ/ppm: −12.7, −144.3 (septet, *J*_PF_ = 707 Hz). ESI MS: *m/z* 968.26 [M − PF_6_]^+^ (calc. 968.27), 811.16 [Cu(xantphos)(Mebpy)]^+^ (base peak calc. 811.21), 641.09 [Cu(xantphos)]^+^ (calc. 641.12). UV-Vis (CH_2_Cl_2_, 2.5 × 10^–5^ mol dm^–3^): λ/nm (ε/dm^3^ mol^–1^ cm^–1^) 248 (50,000), 283 (30,000), 385 (3000). Found: C 63.24, H 4.63, N 6.19; C_59_H_49_CuF_6_N_5_OP_3_ requires C 63.58, H 4.43, N 6.28%.

### 3.9. Crystallography

Single crystal data were collected on a Bruker APEX-II diffractometer with data reduction, solution and refinement using the programs APEX [27] and CRYSTALS [28]. The program CSD Mercury 2020.1 [29] was used for the structure analysis and structural figures. SQUEEZE [30] was used to treat the solvent region, and the electron density removed equated to half a molecule of Et_2_O per complex cation. The anion in [Cu(xantphos)(**3**)][PF_6_]**^.^**0.5Et_2_O was orientationally disordered and was modelled over two positions with fractional occupancies of 0.70 and 0.30. The phenyl ring in ligand **3** was also orientationally disordered over two positions, and the rings had to be refined isotropically and with rigid body restraints.

C_61_H_54_CuF_6_N_5_O_1.5_P_3_, *M*_r_ = 1151.59, yellow block, triclinic, space group *P*–1, *a* = 10.9834(7), *b* = 15.5458(9), *c* = 18.6079(11) Å, α = 110.395(2), β = 94.433(2), γ *=* 110.363(2)^o^, *V* = 2719.7(3) Å^3^, *D*_c_ = 1.41 g cm^–3^, *T* = 123 K, *Z* = 2, μ(CuKα) = 1.981 mm^–1^. Total 36,022 reflections, 10,070 unique (*R*_int_ = 0.029). Refinement of 9303 reflections (682 parameters) with *I* > 2*σ*(*I*) converged at final *R*_1_ = 0.0628 (*R*_1_ all data = 0.0660), *wR*_2_ = 0.1619 (*wR*_2_ all data = 0.1626), gof = 1.0476. CCDC 2047805.

## 4. Conclusions

We have prepared and characterized two new bpy ligands, **2** and **3**, which contain extended 6-substituents. These and ligand **1** have been incorporated into heteroleptic copper(I) coordination compounds [Cu(POP)(**1**)][PF_6_], [Cu(xantphos)(**1**)][PF_6_], [Cu(POP)(**2**)] [PF_6_], [Cu(xantphos)(**2**)][PF_6_], and [Cu(xantphos)(**3**)][PF_6_]. Characterization of these compounds included the determination of the single-crystal structure of [Cu(xantphos)(**3**)][PF_6_]**^.^**0.5Et_2_O, and this confirmed the expected distorted tetrahedral copper(I) coordination environment. The 6-substituent is oriented so that the α- and β-CH_2_ units reside in the xanthene ‘bowl’ of the xantphos ligand, and the conformation of the chain is such that is desymmetrizes the structure. This has implications for the interpretation of the solution NMR spectra of the five complexes, and analysis of the 2D spectra provides evidence for different combinations of possible dynamic processes operating in different compounds. Each copper(I) complex exhibits a broad MLCT absorption band with *λ*_max_ in the range 381–384 nm, and excitation into this band results in a very weak, orange emission in solution. In the solid state, the heteroleptic complexes exhibit emission maxima between 542 nm and 555 nm, and PLQY values range from 13% for [Cu(POP)(**2**)][PF_6_] to 28% for [Cu(xantphos)(**1**)][PF_6_]. These quantum yields are not significantly lower than that of [Cu(xantphos)(6-Mebpy)][PF_6_] and the decay lifetimes of the new compounds are also similar to that of the analogous 6-Mebpy containing derivative. These results demonstrate that going from a 6-methyl to longer-chain substituent is not unfavourable in terms of the photophysical properties.

## Figures and Tables

**Table 1:** Selected bond lengths and angles in the [Cu(xantphos)(**3**)]^+^ cation. See Figure S5 (Supplementary Materials) for atom numbering.

Bond	Bond Length/Å	Angle	Bond Angle/^o^
Cu1–P1	2.2673(7)	P1–Cu1–P2	114.33(3)
Cu1–P2	2.2591(7)	P1–Cu1–N1	113.50(7)
Cu1–N1	2.063(2)	P2–Cu1–N1	111.67(7)
Cu1–N2	2.066(2)	P1–Cu1–N2	113.80(7)
O1–C20	1.384(3)	P2–Cu1–N2	118.78(7)
O1–C22	1.388(3)	N1–Cu1–N2	80.18(10)
N3–N4	1.332(5)	C20–O1–C22	114.96(19)
N4–N5	1.316(5)	N3–N4–N5	108.8(3)

**Table 2:** Copper(I)/(II) oxidation potentials in the copper(I) compounds. Propylene carbonate solutions (ca. 10^−4^ mol dm^–3^); values are referenced internally to Fc/Fc^+^ = 0.0 V; [*^n^*Bu_4_N][PF_6_] as supporting electrolyte and scan rate of 0.1 V s^–1^.

Compound	*E*_pc_^a^/V	*E*_1/2_/V	*E*_pc_ − *E*_pa_/mV
[Cu(POP)(**1**)][PF_6_]	+0.85	−2.07	80
[Cu(xantphos)(**1**)][PF_6_]	+0.93	−2.06	90
[Cu(POP)(**2**)][PF_6_]	+0.92	−2.10	120
[Cu(xantphos)(**2**)][PF_6_]	+0.94	−2.13	130
[Cu(xantphos)(**3**)][PF_6_]	+0.81	−2.02	90

^a^ The value *E*_pc_ is given rather than *E*_1/2_^ox^ because each oxidative process is irreversible.

**Table 3:** Absorption maxima for CH_2_Cl_2_ solutions of the copper(I) compounds (2.5 × 10^−5^ mol dm^−3^).

Compound	*λ*_max_/nm (*ε*_max_/dm^3^ mol^−1^ cm^−1^)	
	π*←π	MLCT
[Cu(POP)(**1**)][PF_6_]	247 sh (28,500), 292 (23,400), 299 sh (22,300), 312 sh (14,800)	381 (2900)
[Cu(xantphos)(**1**)][PF_6_]	247 sh (31,400), 285 (27,700), 315 sh (11,500)	383 (2900)
[Cu(POP)(**2**)][PF_6_]	250 sh (25,600), 292 (21,900), 300 sh (20,500), 312 sh (14,800)	384 (2850)
[Cu(xantphos)(**2**)][PF_6_]	248 sh (26,200), 285 (24,800), 312 sh (10,700)	382 (2700)
[Cu(xantphos)(**3**)][PF_6_]	247 (49,400), 282 (30,600), 312 sh (12,400)	383 (3100)

**Table 4:** Emission properties of the [Cu(P^P)(N^N)][PF_6_] compounds in the solid state.

Compound	*λ*_exc_ /nm	*λ*_max_^em^/ nm	PLQY/%	τ/μs ^a^	τ(1)/μs (A_1_)	τ(2)/μs (A_2_)
[Cu(POP)(**1**)][PF_6_]	365	552	17	5.4	5.94 (0.82)	2.30 (0.14)
[Cu(xantphos)(**1**)][PF_6_]	365	542	28	7.2	8.29 (0.77)	2.16 (0.88)
[Cu(POP)(**2**)][PF_6_]	365	555	13	5.1	5.47 (0.85)	1.88 (0.09)
[Cu(xantphos)(**2**)][PF_6_]	365	546	26	8.7	9.64 (0.83)	2.26 (0.12)
[Cu(xantphos)(**3**)][PF_6_]	365	548	25	7.1	8.64 (0.71)	2.24 (0.22)

^a^A biexponential fit to the lifetime decay was used because a single exponential gave a poor fit; τ is calculated from τ = τ = $\sqrt{\sum A_{i}\tau_{i}/\sum A_{i}}$ where *A_i_* is the pre-exponential factor for the lifetime; values of τ(1), τ(2), A_1_ and A_2_ are given in the right-hand columns of the table.

## Data Availability

The data presented in this study are available on request from the corresponding author. The data are not publicly accessible at present.

## Supplementary Materials

The following are available online, Figures S1–S4: NMR spectra of ligands **2** and **3**; Figures S5 and S6: Structural figures of the [Cu(xantphos)(**3**)]^+^ cation; Figures S7–S23: NMR spectra of the heteroleptic complexes; Figure S24: Cyclic voltammograms of [Cu(POP)(**2**)][PF_6_] and [Cu(xantphos)(**2**)][PF_6_].

## Sample Availability

Samples of the compounds are not available from the authors.
