# Supplementary material for: Desymmetrizing Heteroleptic [Cu(P^P)(N^N)][PF6] Compounds: Effects on Structural and Photophysical Properties, and Solution Dynamic Behavior
Source: Molecules. 2020 Dec 29;26(1):125. doi: 10.3390/molecules26010125 (PMC7796056; doi:10.3390/molecules26010125)

checkCIF/PLATON report

Structure factors have been supplied for datablock(s) BRF160_123K

THIS REPORT IS FOR GUIDANCE ONLY. IF USED AS PART OF A REVIEW PROCEDURE FOR PUBLICATION, IT SHOULD NOT REPLACE THE EXPERTISE OF AN EXPERIENCED CRYSTALLOGRAPHIC REFEREE.

No syntax errors found. CIF dictionary Interpreting this report

Datablock: BRF160_123K

Bond precision: C-C = 0.0051 A Wavelength=1.54180

Cell:	a=10.9834(7)	b=15.5458(9)	c=18.6079(11)
	alpha=110.395(2)	beta=94.433(2)	gamma=110.363(2)
Temperature:	123 K		

	Calculated	Reported
Volume	2719.7(3)	2719.7(3)
Space group	P -1	P -1
Hall group	-P 1	-P 1
Moiety formula	C59 H49 Cu N5 O P2, F6 P [+ solvent]	[C59 H49 CU1 N5 O1 P2][PF6] 0.5(ET2O)
Sum formula	C59 H49 Cu F6 N5 O P3 [+ solvent]	C61 H54 Cu1 F6 N5 O1.50 P3
Mr	1114.49	1151.59
Dx,g cm-3	1.361	1.410
Z	2	2
Mu (mm-1)	1.956	1.981
F000	1148.0	1190.0
F000’	1149.08	
h,k,lmax	13,18,22	13,18,22
Nref	10371	10070
Tmin,Tmax	0.788,0.906	0.770,0.910
Tmin’	0.788	

Correction method= # Reported T Limits: Tmin=0.770 Tmax=0.910
AbsCorr = MULTI-SCAN

Data completeness= 0.971 Theta(max)= 70.359

R(reflections)= 0.0628(9303) wR2(reflections)= 0.1626(10017)

S = 1.048 Npar= 682

The following ALERTS were generated. Each ALERT has the format
test-name_ALERT_alert-type_alert-level.
Click on the hyperlinks for more details of the test.

Alert level B

```
PLAT201_ALERT_2_B Isotropic non-H Atoms in Main Residue(s) .......          6 Report
PLAT910_ALERT_3_B Missing # of FCF Reflection(s) Below Theta(Min).         46 Note
```

Alert level C

```
CHEMW03_ALERT_2_C The ratio of given/expected molecular weight as
            calculated from the _atom_site* data lies outside
            the range 0.99 <> 1.01
           From the CIF: _cell_formula_units_Z                    2
           From the CIF: _chemical_formula_weight          1151.59
           TEST: Calculate formula weight from _atom_site_*
           atom     mass    num     sum
           C        12.01   59.00  708.65
           H         1.01   49.00   49.39
           N        14.01    5.00   70.04
           O        16.00    1.00   16.00
           F        19.00    6.00  113.99
           P        30.97    3.00   92.92
           Cu       63.55    1.00   63.55
           Calculated formula weight             1114.53
PLAT094_ALERT_2_C Ratio of Maximum / Minimum Residual Density ....       2.28 Report
PLAT220_ALERT_2_C Non-Solvent  Resd 1  C   Ueq(max)/Ueq(min) Range        5.4 Ratio
PLAT222_ALERT_3_C Non-Solv.  Resd 1  H   Uiso(max)/Uiso(min) Range        5.2 Ratio
PLAT241_ALERT_2_C High   'MainMol' Ueq as Compared to Neighbors of        C56 Check
PLAT330_ALERT_2_C Large Average Phenyl C-C Dist C2       -C24            1.42 Ang.
PLAT911_ALERT_3_C Missing FCF Refl Between Thmin & STh/L=    0.600         79 Report
PLAT918_ALERT_3_C Reflection(s) with I(obs) much Smaller I(calc) .          2 Check
```

Alert level G

```
FORMU01_ALERT_1_G  There is a discrepancy between the atom counts in the
            _chemical_formula_sum and _chemical_formula_moiety. This is
            usually due to the moiety formula being in the wrong format.
            Atom count from _chemical_formula_sum:   C61 H54 Cu1 F6 N5 O1.5 P3
            Atom count from _chemical_formula_moiety:
FORMU01_ALERT_2_G  There is a discrepancy between the atom counts in the
            _chemical_formula_sum and the formula from the _atom_site* data.
            Atom count from _chemical_formula_sum:C61 H54 Cu1 F6 N5 O1.5 P3
            Atom count from the _atom_site data:  C59 H49 Cu1 F6 N5 O1 P3
CELLZ01_ALERT_1_G Difference between formula and atom_site contents detected.
CELLZ01_ALERT_1_G ALERT: Large difference may be due to a
            symmetry error - see SYMMG tests
           From the CIF: _cell_formula_units_Z    2
           From the CIF: _chemical_formula_sum  C61 H54 Cu1 F6 N5 O1.50 P3
           TEST: Compare cell contents of formula and atom_site data

           atom    Z*formula  cif sites diff
           C        122.00    118.00    4.00
           H        108.00     98.00   10.00
           Cu         2.00      2.00    0.00
           F         12.00     12.00    0.00
           N         10.00     10.00    0.00
           O          3.00      2.00    1.00
           P          6.00      6.00    0.00
PLAT003_ALERT_2_G Number of Uiso or Uij Restrained non-H Atoms ...         81 Report
```

```
PLAT041_ALERT_1_G Calc. and Reported SumFormula    Strings  Differ     Please Check
PLAT042_ALERT_1_G Calc. and Reported MoietyFormula Strings  Differ     Please Check
PLAT068_ALERT_1_G Reported F000 Differs from Calcd (or Missing)...     Please Check
PLAT093_ALERT_1_G No s.u.'s on H-positions, Refinement Reported as      mixed Check
PLAT154_ALERT_1_G The s.u.'s on the Cell Angles are Equal ..(Note)      0.002 Degree
PLAT232_ALERT_2_G Hirshfeld Test Diff (M-X) Cu1      --P1        .        5.5 s.u.
PLAT244_ALERT_4_G Low    'Solvent' Ueq as Compared to Neighbors of         P3 Check
PLAT300_ALERT_4_G Atom Site Occupancy of C2         Constrained at        0.7 Check
PLAT300_ALERT_4_G Atom Site Occupancy of C21        Constrained at        0.7 Check
PLAT300_ALERT_4_G Atom Site Occupancy of C24        Constrained at        0.7 Check
PLAT300_ALERT_4_G Atom Site Occupancy of C44        Constrained at        0.7 Check
PLAT300_ALERT_4_G Atom Site Occupancy of C51        Constrained at        0.7 Check
PLAT300_ALERT_4_G Atom Site Occupancy of C55        Constrained at        0.7 Check
PLAT300_ALERT_4_G Atom Site Occupancy of C1         Constrained at        0.3 Check
PLAT300_ALERT_4_G Atom Site Occupancy of C58        Constrained at        0.3 Check
PLAT300_ALERT_4_G Atom Site Occupancy of C59        Constrained at        0.3 Check
PLAT300_ALERT_4_G Atom Site Occupancy of C70        Constrained at        0.3 Check
PLAT300_ALERT_4_G Atom Site Occupancy of C71        Constrained at        0.3 Check
PLAT300_ALERT_4_G Atom Site Occupancy of C72        Constrained at        0.3 Check
PLAT300_ALERT_4_G Atom Site Occupancy of H21        Constrained at        0.7 Check
PLAT300_ALERT_4_G Atom Site Occupancy of H241       Constrained at        0.7 Check
PLAT300_ALERT_4_G Atom Site Occupancy of H441       Constrained at        0.7 Check
PLAT300_ALERT_4_G Atom Site Occupancy of H511       Constrained at        0.7 Check
PLAT300_ALERT_4_G Atom Site Occupancy of H551       Constrained at        0.7 Check
PLAT300_ALERT_4_G Atom Site Occupancy of H11        Constrained at        0.3 Check
PLAT300_ALERT_4_G Atom Site Occupancy of H581       Constrained at        0.3 Check
PLAT300_ALERT_4_G Atom Site Occupancy of H701       Constrained at        0.3 Check
PLAT300_ALERT_4_G Atom Site Occupancy of H711       Constrained at        0.3 Check
PLAT300_ALERT_4_G Atom Site Occupancy of H721       Constrained at        0.3 Check
PLAT300_ALERT_4_G Atom Site Occupancy of F1         Constrained at        0.7 Check
PLAT300_ALERT_4_G Atom Site Occupancy of F2         Constrained at        0.7 Check
PLAT300_ALERT_4_G Atom Site Occupancy of F3         Constrained at        0.7 Check
PLAT300_ALERT_4_G Atom Site Occupancy of F6         Constrained at        0.7 Check
PLAT300_ALERT_4_G Atom Site Occupancy of F7         Constrained at        0.3 Check
PLAT300_ALERT_4_G Atom Site Occupancy of F8         Constrained at        0.3 Check
PLAT300_ALERT_4_G Atom Site Occupancy of F9         Constrained at        0.3 Check
PLAT300_ALERT_4_G Atom Site Occupancy of F10        Constrained at        0.3 Check
PLAT301_ALERT_3_G Main Residue  Disorder .............(Resd  1  )         9% Note
PLAT302_ALERT_4_G Anion/Solvent/Minor-Residue Disorder (Resd  2  )        57% Note
PLAT605_ALERT_4_G Largest Solvent Accessible VOID in the Structure         74 A**3
PLAT808_ALERT_5_G No Parseable SHELXL Style Weighting Scheme Found     Please Check
PLAT860_ALERT_3_G Number of Least-Squares Restraints .............        708 Note
PLAT869_ALERT_4_G ALERTS Related to the Use of SQUEEZE Suppressed           ! Info
PLAT882_ALERT_1_G No Datum for _diffrn_reflns_av_unetI/netI ......     Please Do !
PLAT912_ALERT_4_G Missing # of FCF Reflections Above STh/L=  0.600        230 Note
PLAT929_ALERT_5_G No Weight Pars,Obs and Calc R1,wR2,S not Checked          ! Info
PLAT960_ALERT_3_G Number of Intensities with I  <   - 2*sig(I) ...          3 Check
```

```
  0 ALERT level A = Most likely a serious problem - resolve or explain
  2 ALERT level B = A potentially serious problem, consider carefully
  8 ALERT level C = Check. Ensure it is not caused by an omission or oversight
 52 ALERT level G = General information/check it is not something unexpected

  9 ALERT type 1 CIF construction/syntax error, inconsistent or missing data
  9 ALERT type 2 Indicator that the structure model may be wrong or deficient
  7 ALERT type 3 Indicator that the structure quality may be low
 35 ALERT type 4 Improvement, methodology, query or suggestion
  2 ALERT type 5 Informative message, check
```

Validation response form

Please find below a validation response form (VRF) that can be filled in and pasted into your CIF.

```
# start Validation Reply Form
_vrf_CHEMW03_BRF160_123K
;
PROBLEM: The ratio of given/expected molecular weight as
RESPONSE: ...
;
_vrf_PLAT094_BRF160_123K
;
PROBLEM: Ratio of Maximum / Minimum Residual Density ....       2.28 Report
RESPONSE: ...
;
_vrf_PLAT220_BRF160_123K
;
PROBLEM: Non-Solvent  Resd 1  C   Ueq(max)/Ueq(min) Range        5.4 Ratio
RESPONSE: ...
;
_vrf_PLAT222_BRF160_123K
;
PROBLEM: Non-Solv.  Resd 1  H   Uiso(max)/Uiso(min) Range        5.2 Ratio
RESPONSE: ...
;
_vrf_PLAT241_BRF160_123K
;
PROBLEM: High   'MainMol' Ueq as Compared to Neighbors of        C56 Check
RESPONSE: ...
;
_vrf_PLAT330_BRF160_123K
;
PROBLEM: Large Average Phenyl C-C Dist C2       -C24            1.42 Ang.
RESPONSE: ...
;
_vrf_PLAT911_BRF160_123K
;
PROBLEM: Missing FCF Refl Between Thmin & STh/L=    0.600         79 Report
RESPONSE: ...
;
_vrf_PLAT918_BRF160_123K
;
PROBLEM: Reflection(s) with I(obs) much Smaller I(calc) .          2 Check
RESPONSE: ...
;
# end Validation Reply Form
```

It is advisable to attempt to resolve as many as possible of the alerts in all categories. Often the minor alerts point to easily fixed oversights, errors and omissions in your CIF or refinement strategy, so attention to these fine details can be worthwhile. In order to resolve some of the more serious problems it may be necessary to carry out additional measurements or structure refinements. However, the purpose of your study may justify the reported deviations and the more serious of these should normally be commented upon in the discussion or experimental section of a paper or in the "special_details" fields of the CIF. checkCIF was carefully designed to identify outliers and unusual parameters, but every test has its limitations and alerts that are not important in a particular case may appear. Conversely, the absence of alerts does not guarantee there are no aspects of the results needing attention. It is up to the individual to critically assess their own results and, if necessary, seek expert advice.

Publication of your CIF in IUCr journals

A basic structural check has been run on your CIF. These basic checks will be run on all CIFs submitted for publication in IUCr journals (*Acta Crystallographica*, *Journal of Applied Crystallography*, *Journal of Synchrotron Radiation*); however, if you intend to submit to *Acta Crystallographica Section C* or *E* or *IUCrData*, you should make sure that full publication checks are run on the final version of your CIF prior to submission.

Publication of your CIF in other journals

Please refer to the *Notes for Authors* of the relevant journal for any special instructions relating to CIF submission.

PLATON version of 23/04/2018; check.def file version of 23/04/2018

Datablock BRF160_123K - ellipsoid plot

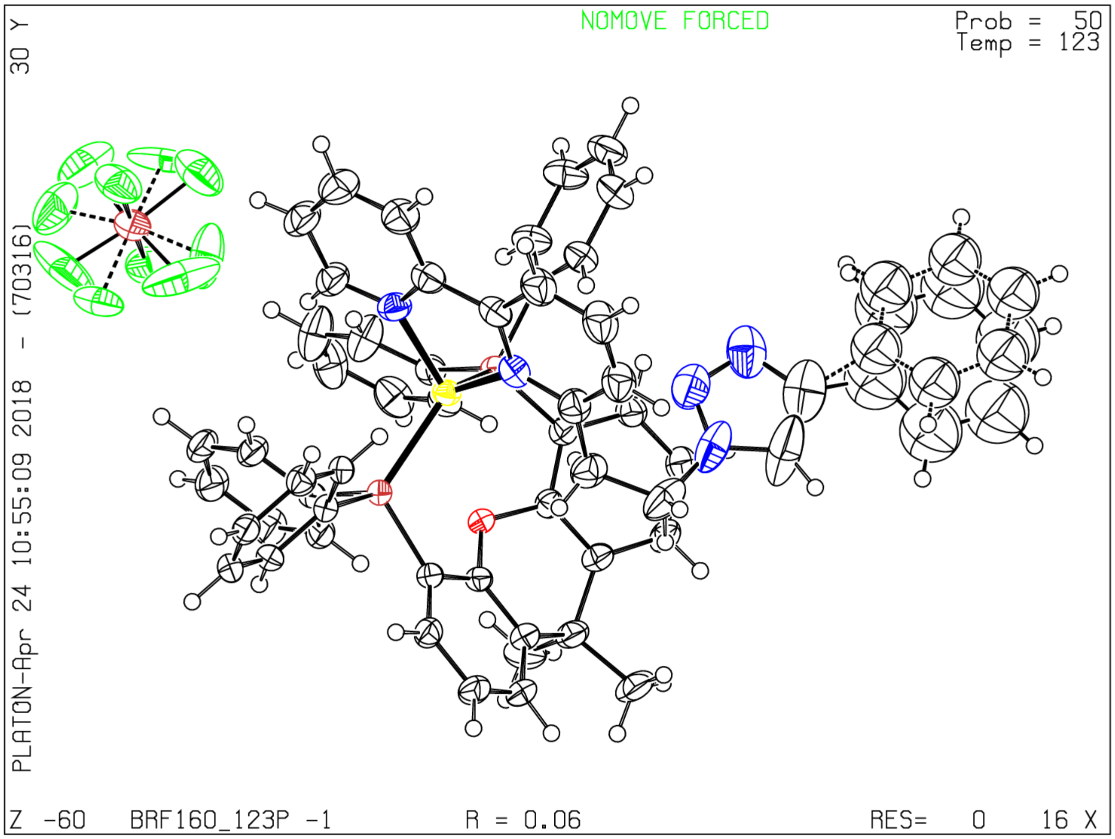

Supplement: Supplementary file 1 [file molecules-26-00125-s001.zip › molecules-1056582-SI/supporting info/checkcif.pdf]
