# Supplementary material for: Desymmetrizing Heteroleptic [Cu(P^P)(N^N)][PF6] Compounds: Effects on Structural and Photophysical Properties, and Solution Dynamic Behavior
Source: Molecules. 2020 Dec 29;26(1):125. doi: 10.3390/molecules26010125 (PMC7796056; doi:10.3390/molecules26010125)
Supplement: Supplementary file 1 [file molecules-26-00125-s001.zip › molecules-1056582-SI/supporting info/Supporting Material.pdf]

Supporting Material

Desymmetrizing heteroleptic [Cu(P^P)(N^N)][PF_6] compounds: effects on structural, dynamic and photophysical properties

Marco Meyer, Fabian Brunner, Alessandro Prescimone, Edwin C. Constable and Catherine E. Housecroft*

Department of Chemistry, University of Basel, BPR 1096, Mattenstrasse 24a, CH-4058 Basel, Switzerland

Table of contents

Figure S1. HMQC spectrum of **2**.
Figure S2. HMBC spectrum of **2**.
Figure S3. HMQC spectrum of **3**.
Figure S4. HMBC spectrum of **3**.
Figure S5. ORTEP representation of the structure of the [Cu(xantphos)(**3**)]$^+$ cation.
Figure S6. π-Stacking interaction in the [Cu(xantphos)(**3**)]$^+$ cation.
Figure S7. ^{1}H NMR spectrum of [Cu(POP)(**1**)][PF_6].
Figure S8. HMQC spectrum of [Cu(POP)(**1**)][PF_6].
Figure S9. HMBC spectrum of [Cu(POP)(**1**)][PF_6].
Figure S10. Part of the ^{1}H NMR spectrum of [Cu(xantphos)(**1**)][PF_6].
Figure S11. Part of the HMQC spectrum of [Cu(xantphos)(**1**)][PF_6].
Figure S12. Part of the HMBC spectrum of [Cu(xantphos)(**1**)][PF_6].
Figure S13. Aromatic region of the ^{1}H NMR spectrum of [Cu(POP)(**2**)][PF_6].
Figure S14. Aromatic region of the HMQC spectrum of [Cu(POP)(**2**)][PF_6],
Figure S15. Aromatic region of the HMBC spectrum of [Cu(POP)(**2**)][PF_6]
Figure S16. Aromatic region of the ^{1}H NMR spectrum of [Cu(xantphos)(**2**)][PF_6].
Figure S17. Aromatic region of the HMQC spectrum of [Cu(xantphos)(**2**)][PF_6].
Figure S18. Aromatic region of the HMBC spectrum of [Cu(xantphos)(**2**)][PF_6].
Figure S19. Aromatic region of the ^{1}H NMR spectrum of [Cu(xantphos)(**3**)][PF_6].
Figure S20. Aromatic region of the HMQC spectrum of [Cu(xantphos)(**3**)][PF_6].
Figure S21. Aromatic region of the HMBC spectrum of [Cu(xantphos)(**3**)][PF_6].
Figure S22. Aromatic regions of the ^{1}H NMR spectra of [Cu(POP)(**2**)][PF_6] and [Cu(xantphos)(**2**)][PF_6].
Figure S23. Part of the NOESY spectra of [Cu(xantphos)(**2**)][PF_6] and [Cu(POP)(**2**)][PF_6].
Figure S24. Cyclic voltammograms of [Cu(POP)(**2**)][PF_6] and [Cu(xantphos)(**2**)][PF_6].

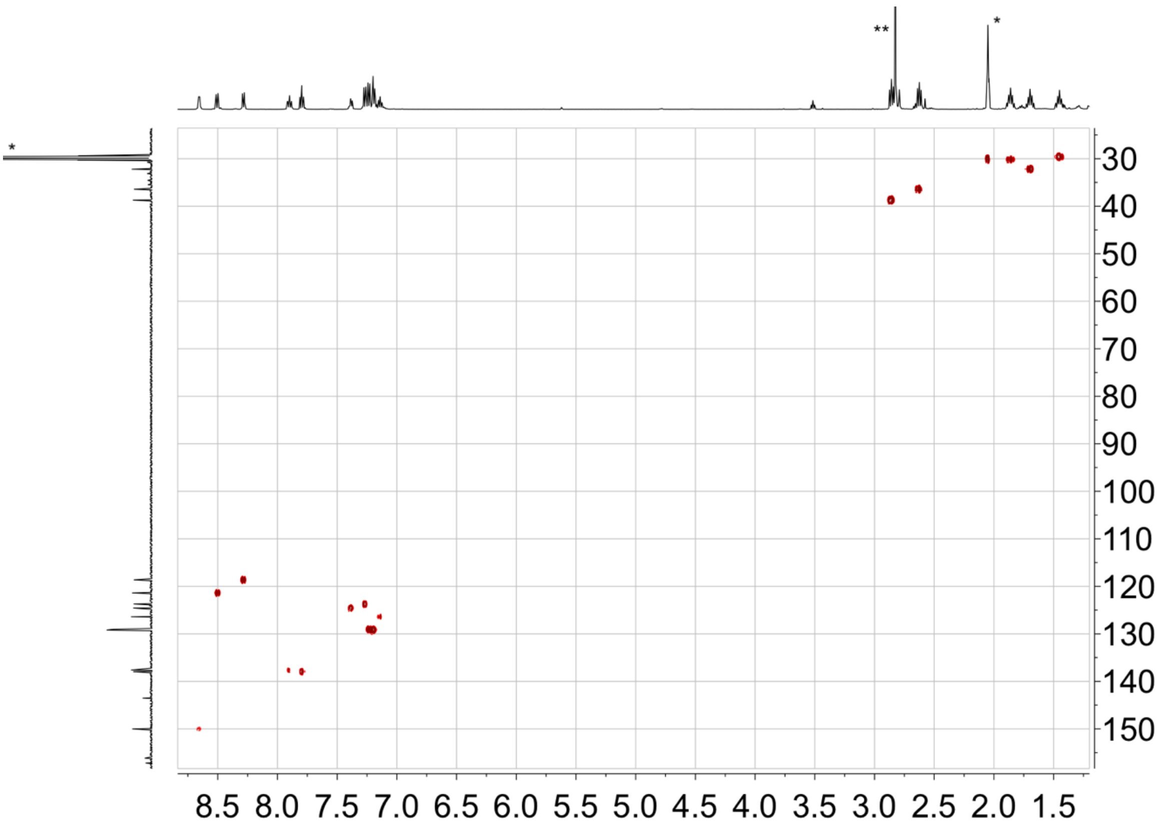

Figure S1. HMQC spectrum of **2** (^{1}H 500 MHz, ^{13}C 126 MHz, acetone-d$_6$, 298 K). * = acetone-d$_6$ (in ^{13}C{^{1}H} or residual acetone-d$_5$ (in ^{1}H); ** = H_2O.

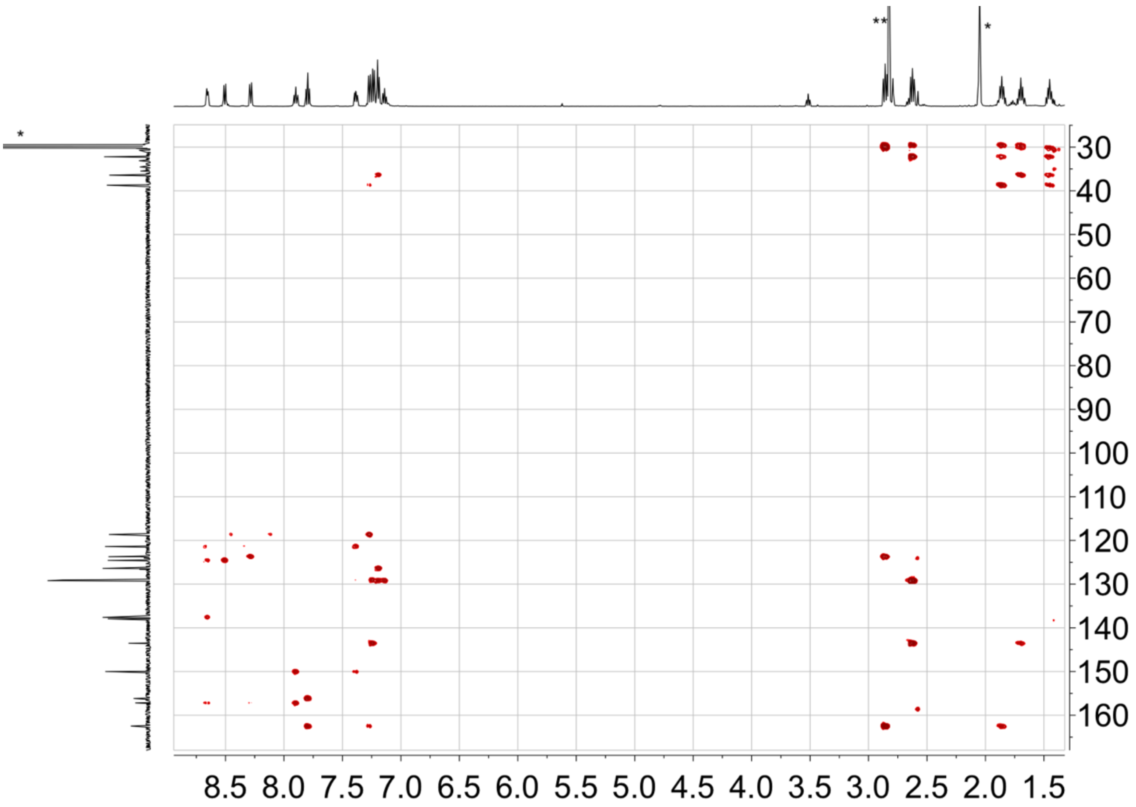

Figure S2. HMBC spectrum of **2** (^{1}H 500 MHz, ^{13}C 126 MHz, acetone-d$_6$, 298 K). * = acetone-d$_6$ (in ^{13}C{^{1}H} or residual acetone-d$_5$ (in ^{1}H); ** = H_2O.

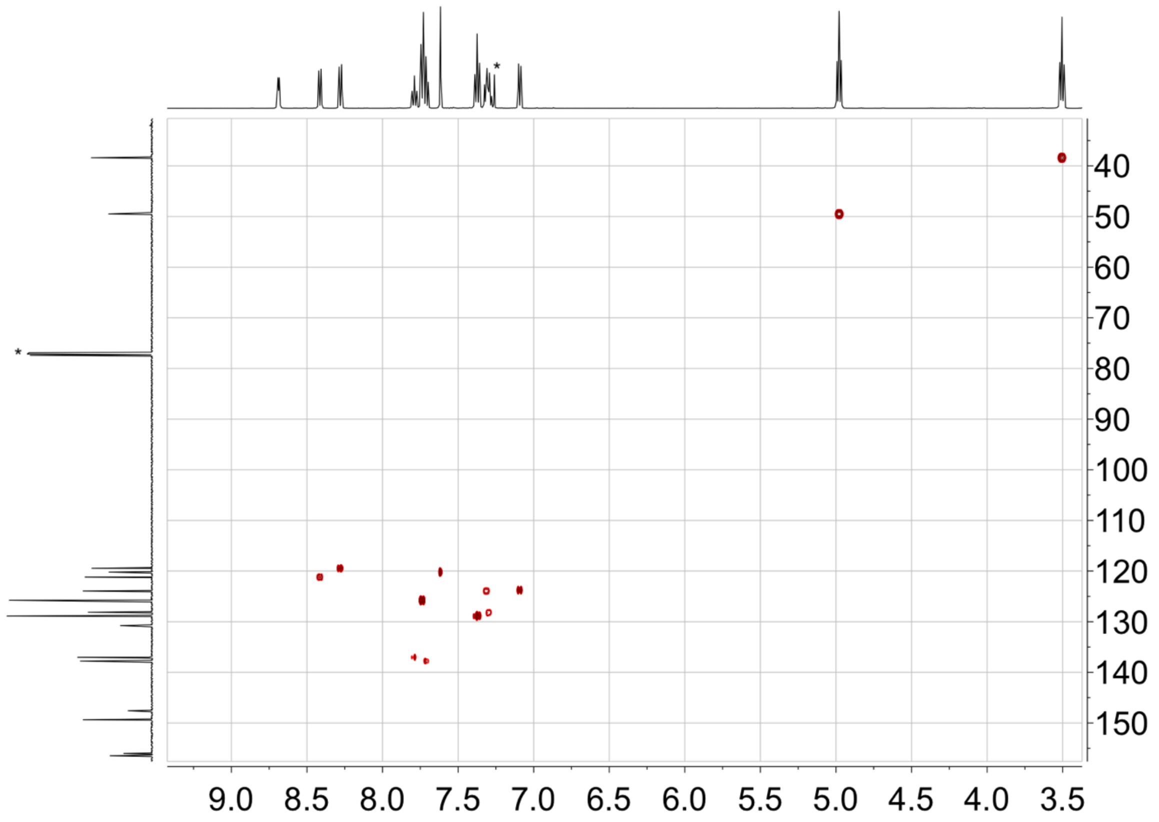

Figure S3. HMQC spectrum of **3** (^1H 500 MHz, ^{13}C 126 MHz,$CDCl_3$, 298 K). * = $CDCl_3$ (in $^{13}C\{^1H\}$ or residual $CHCl_3$ (in ^1H); ** = H_2O.

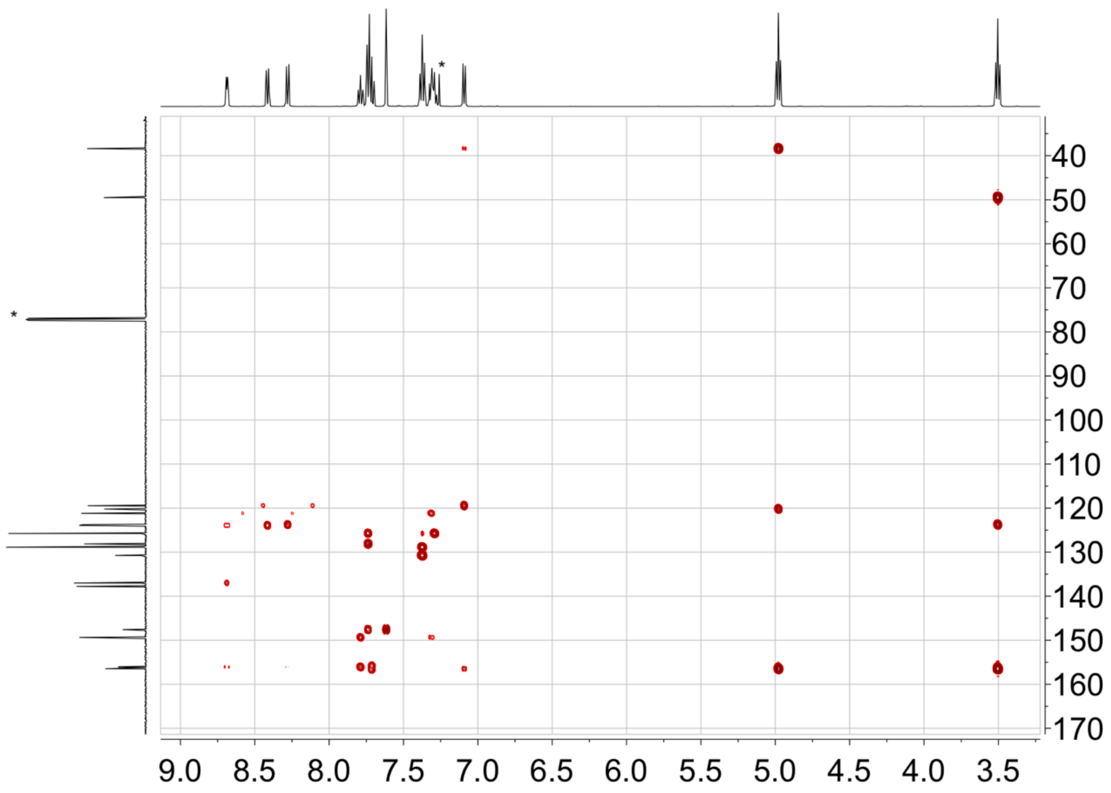

Figure S4. HMBC spectrum of **3** (^1H 500 MHz, ^{13}C 126 MHz, $CDCl_3$, 298 K). * = $CDCl_3$ (in $^{13}C\{^1H\}$ or residual $CHCl_3$ (in ^1H); ** = H_2O.

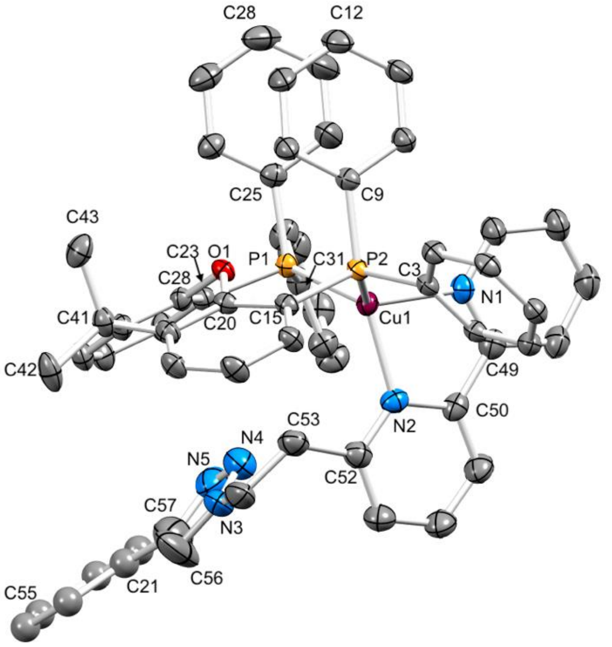

Figure S5. ORTEP representation of the structure of the [Cu(xantphos)(**3**)]$^+$ cation in [Cu(xantphos)(**3**)][PF_6]·0.5Et_2O. Ellipsoids are drawn at a 40% probability level and H atoms are omitted; the phenyl ring in ligand **3** was orientationally disordered and the rings were refined isotropically.

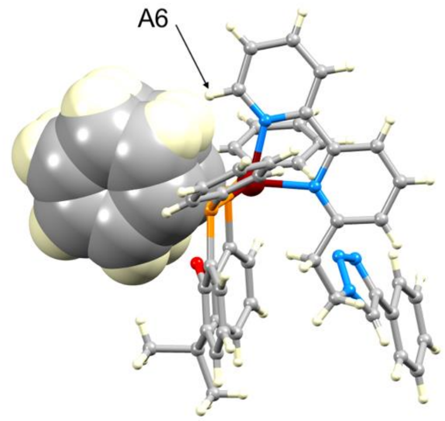

Figure S6. π-Stacking interaction in the [Cu(xantphos)(**3**)]$^+$ cation and the proximity of proton H^{A6} to these phenyl rings.

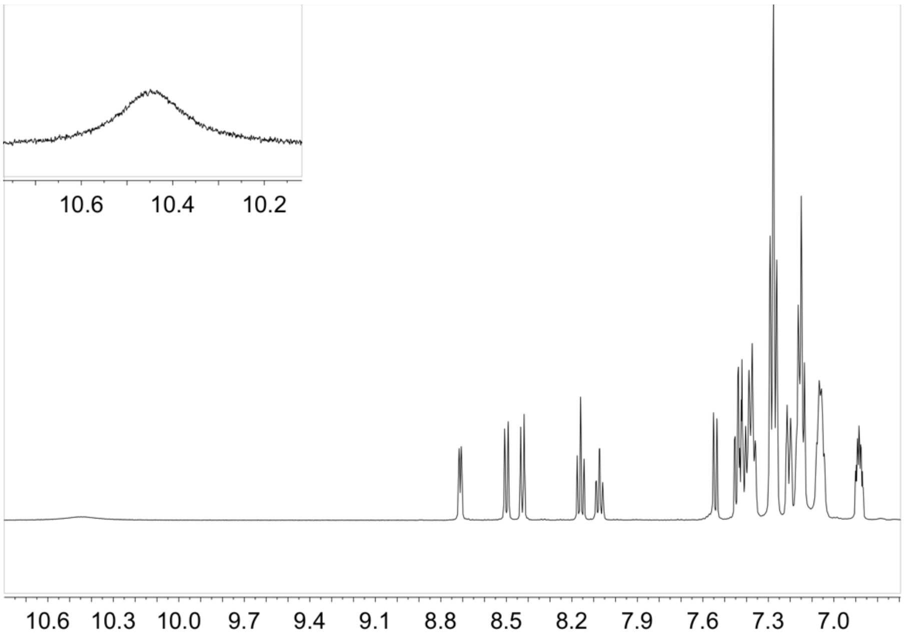

Figure S7. ^{1}H NMR spectrum of [Cu(POP)(**1**)][PF_6] (500 MHz, acetone-d_6, 298 K) with the CO_2H signal inset.

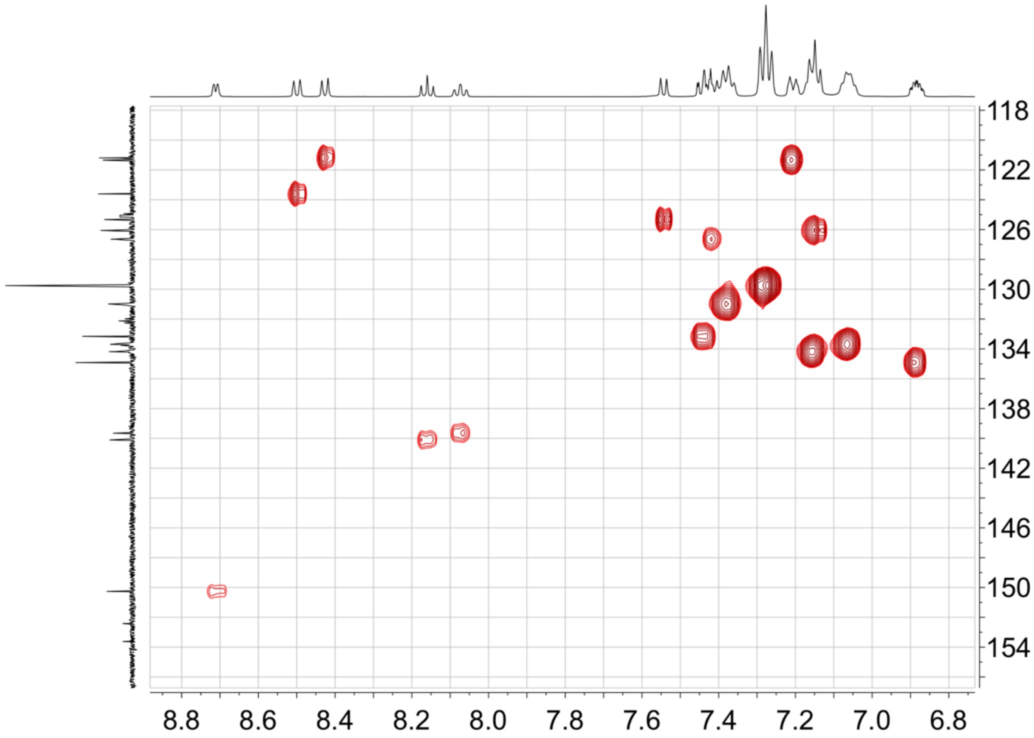

Figure S8. HMQC spectrum of [Cu(POP)(**1**)][PF_6] (^{1}H 500 MHz, ^{13}C 126 MHz, acetone-d_6, 298 K).

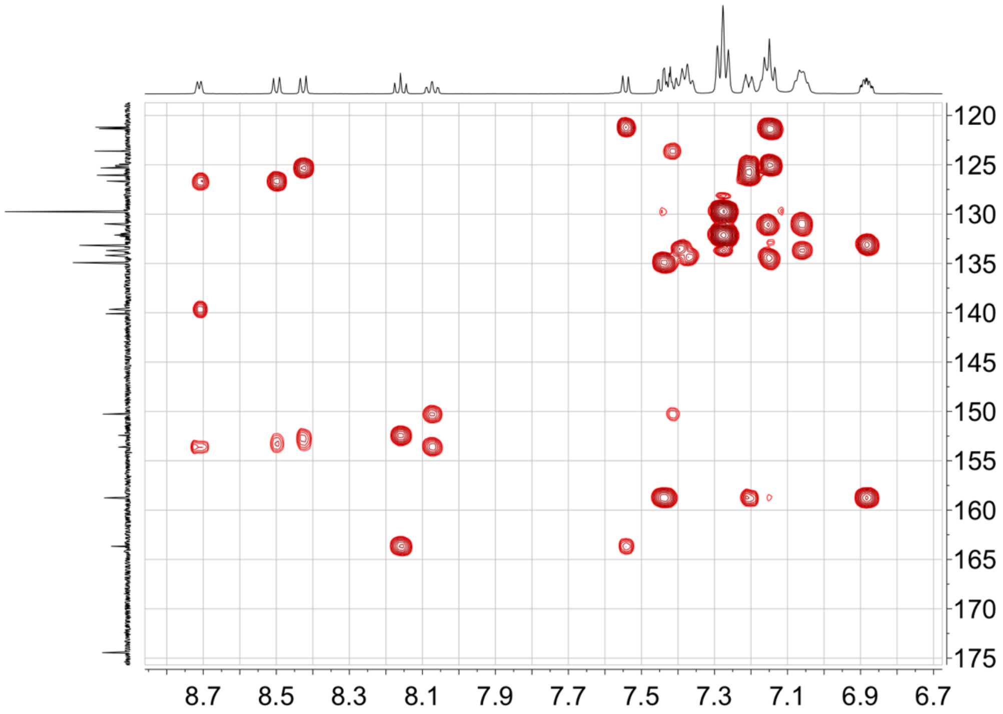

Figure S9. HMBC spectrum of [Cu(POP)(**1**)][PF_6] (^1H 500 MHz, ^{13}C 126 MHz, acetone-d_6, 298 K).

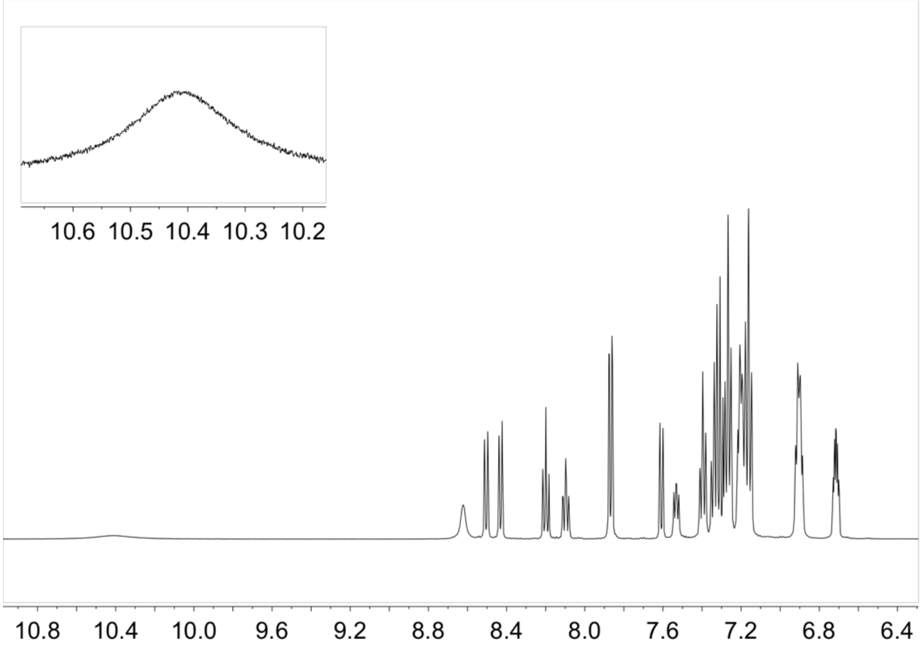

Figure S10. Part of the ^1H NMR spectrum of [Cu(xantphos)(**1**)][PF_6] (500 MHz, acetone-d_6, 298 K) with the CO_2H signal inset.

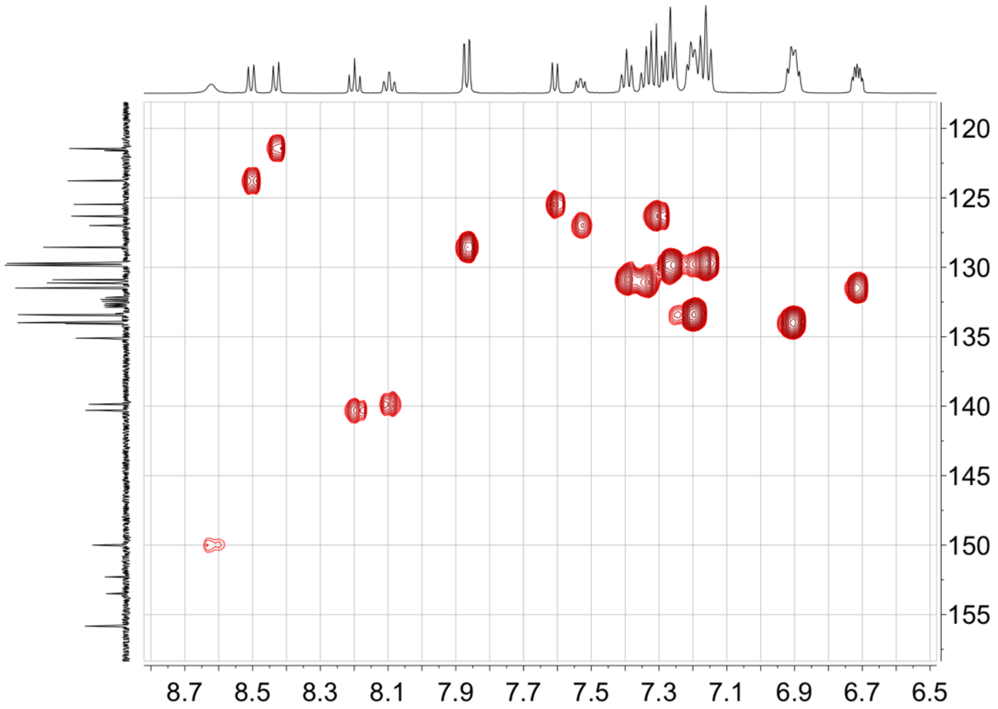

Figure S11. Part of the HMQC spectrum of [Cu(xantphos)(**1**)][PF_6] (^{1}H 500 MHz, ^{13}C 126 MHz, acetone-d_6, 298 K).

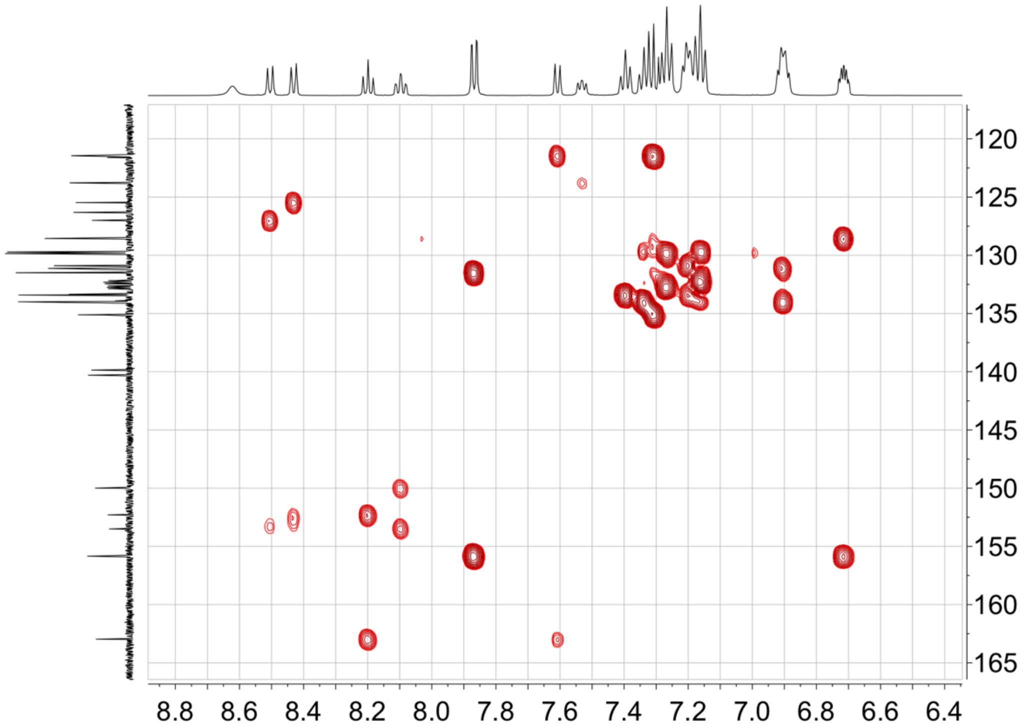

Figure S12. Part of the HMBC spectrum of [Cu(xantphos)(**1**)][PF_6] (^{1}H 500 MHz, ^{13}C 126 MHz, acetone-d_6, 298 K).

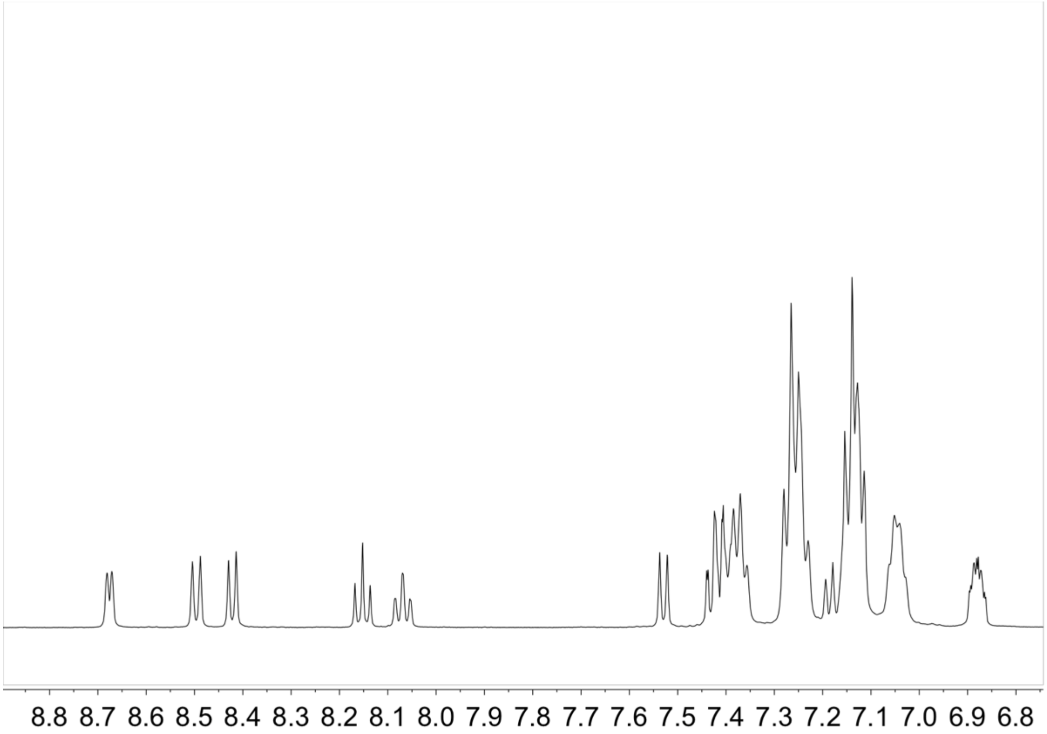

Figure S13. Aromatic region of the ^{1}H NMR spectrum of [Cu(POP)(**2**)][PF_6] (500 MHz, acetone-d_6, 298 K).

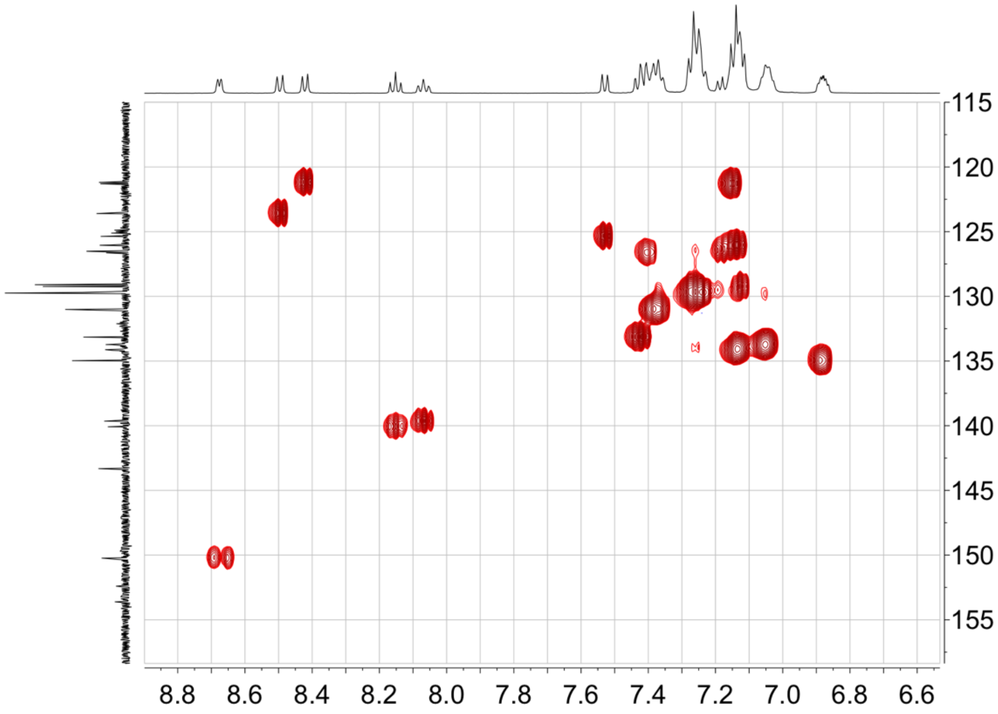

Figure S14. Aromatic region of the HMQC spectrum of [Cu(POP)(**2**)][PF_6] (^{1}H 500 MHz, ^{13}C 126 MHz, acetone-d_6, 298 K).

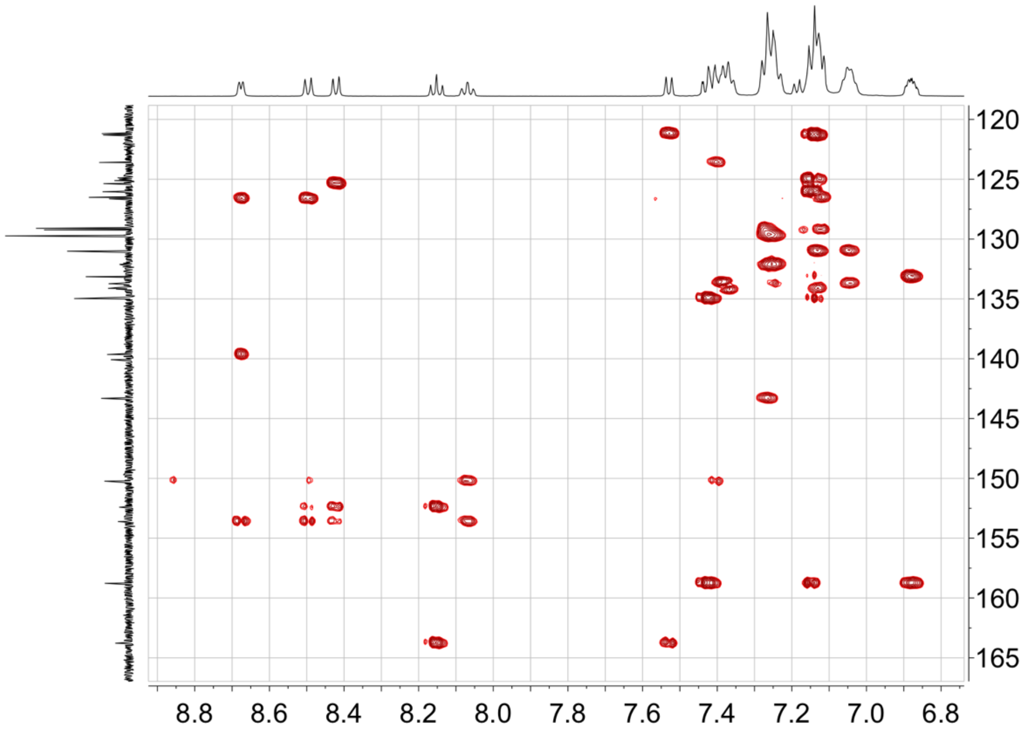

Figure S15. Aromatic region of the HMBC spectrum of [Cu(POP)(**2**)][PF$_6$] (^{1}H 500 MHz, ^{13}C 126 MHz, acetone-d$_6$, 298 K).

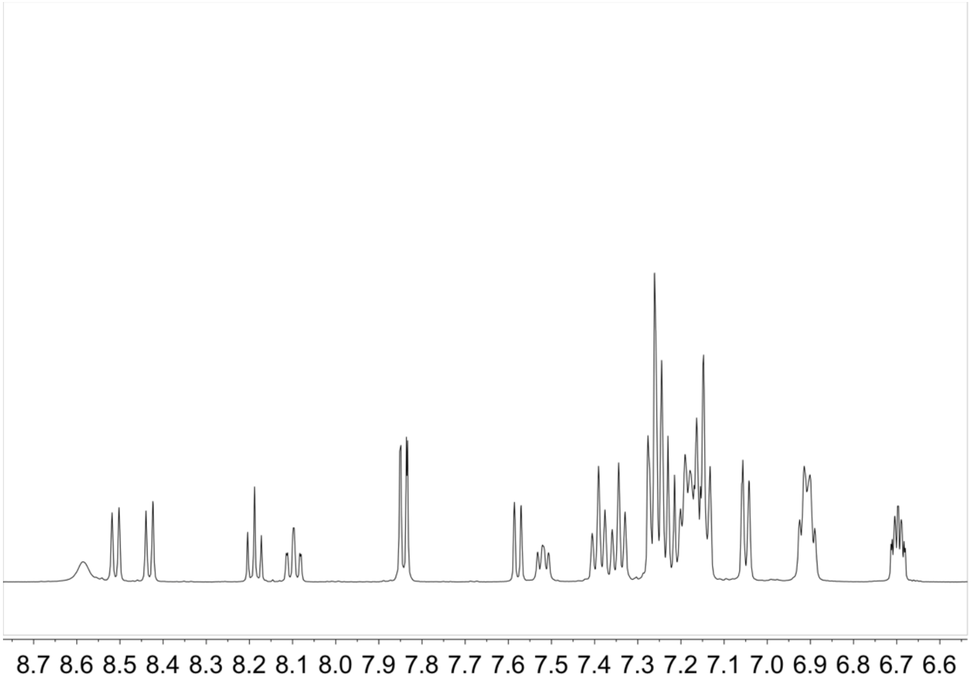

Figure S16. Aromatic region of the ^{1}H NMR spectrum of [Cu(xantphos)(**2**)][PF$_6$] (500 MHz, acetone-d$_6$, 298 K).

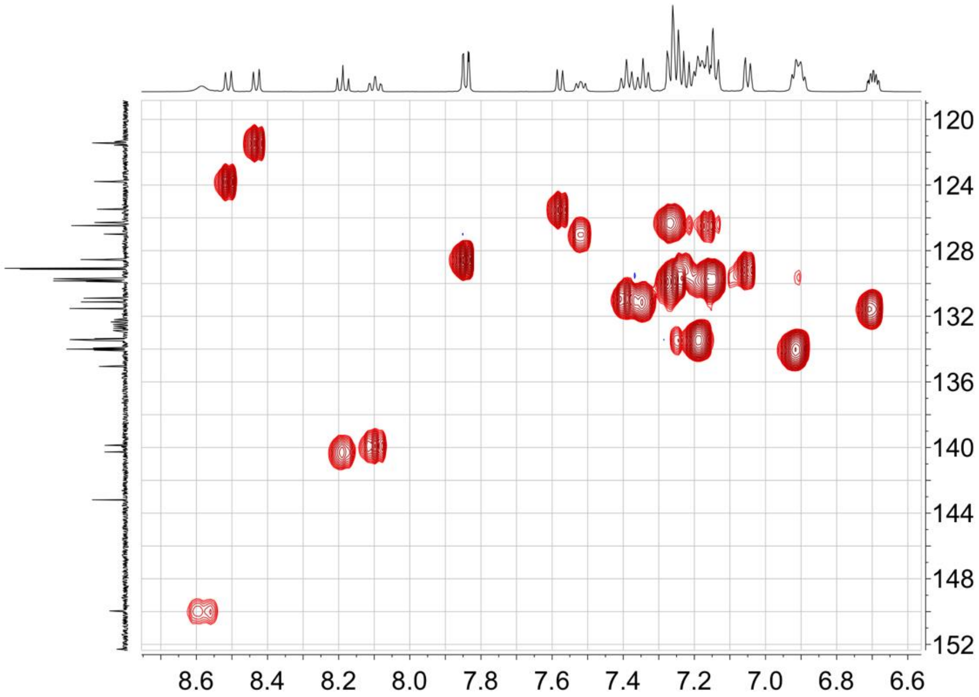

Figure S17. Aromatic region of the HMQC spectrum of [Cu(xantphos)(**2**)][PF_6] (^{1}H 500 MHz, ^{13}C 126 MHz, acetone-d$_6$, 298 K).

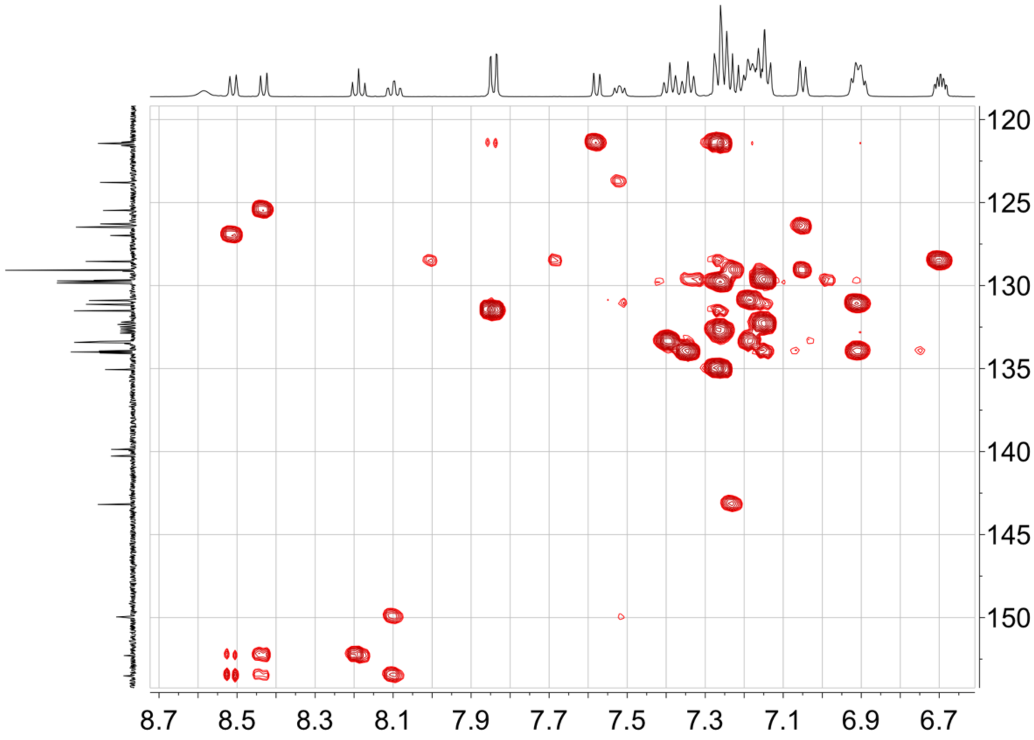

Figure S18. Aromatic region of the HMBC spectrum of [Cu(xantphos)(**2**)][PF_6] (^{1}H 500 MHz, ^{13}C 126 MHz, acetone-d$_6$, 298 K).

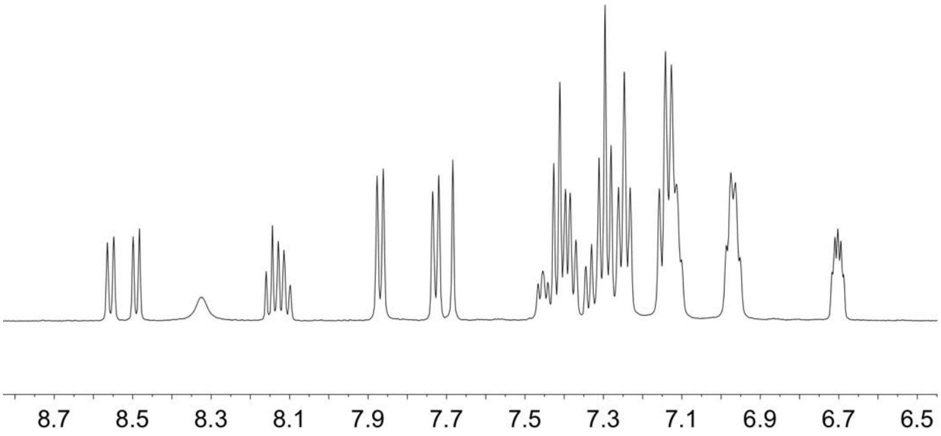

Figure S19. Aromatic region of the ^{1}H NMR spectrum of [Cu(xantphos)(**3**)][PF_6] (500 MHz, acetone-d_6, 298 K).

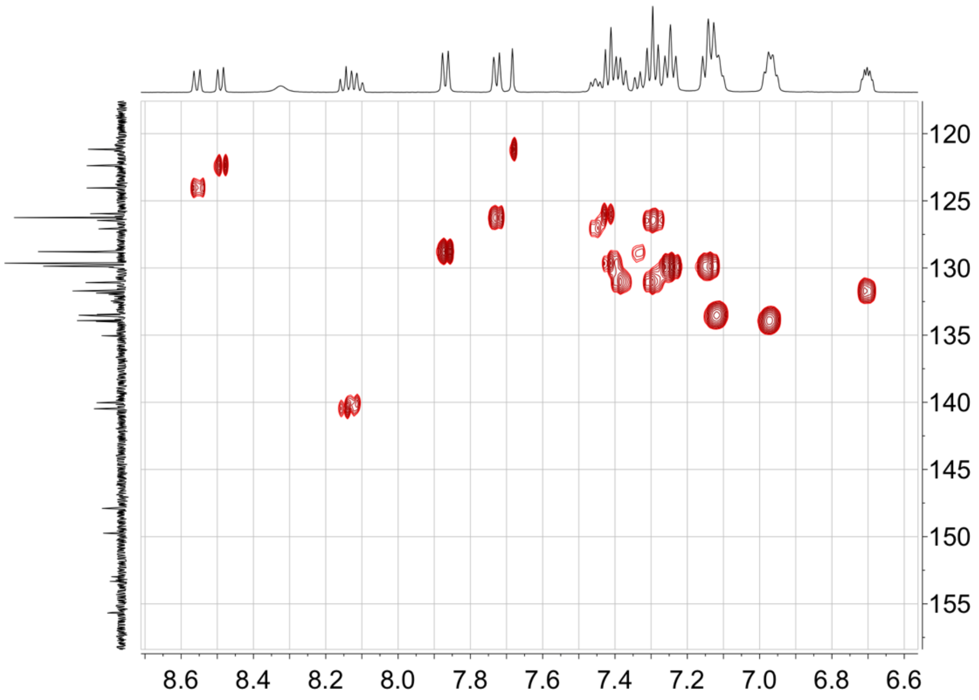

Figure S20. Aromatic region of the HMQC spectrum of [Cu(xantphos)(**3**)][PF_6] (^{1}H 500 MHz, ^{13}C 126 MHz, acetone-d_6, 298 K).

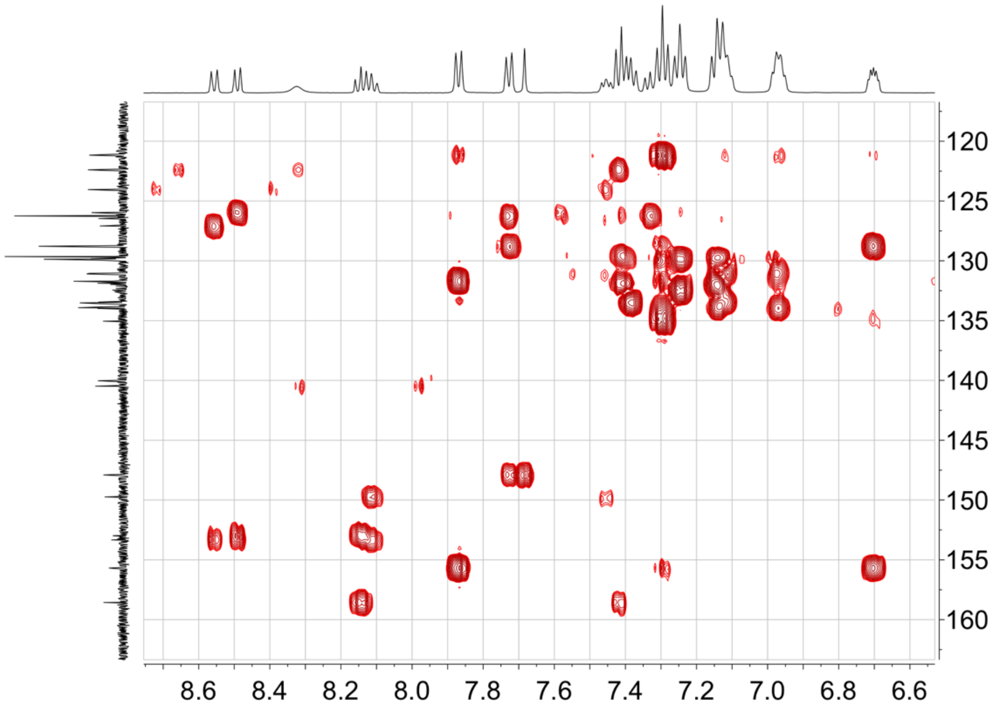

Figure S21. Aromatic region of the HMBC spectrum of [Cu(xantphos)(**3**)][PF$_6$] (^{1}H 500 MHz, ^{13}C 126 MHz, acetone-d$_6$, 298 K).

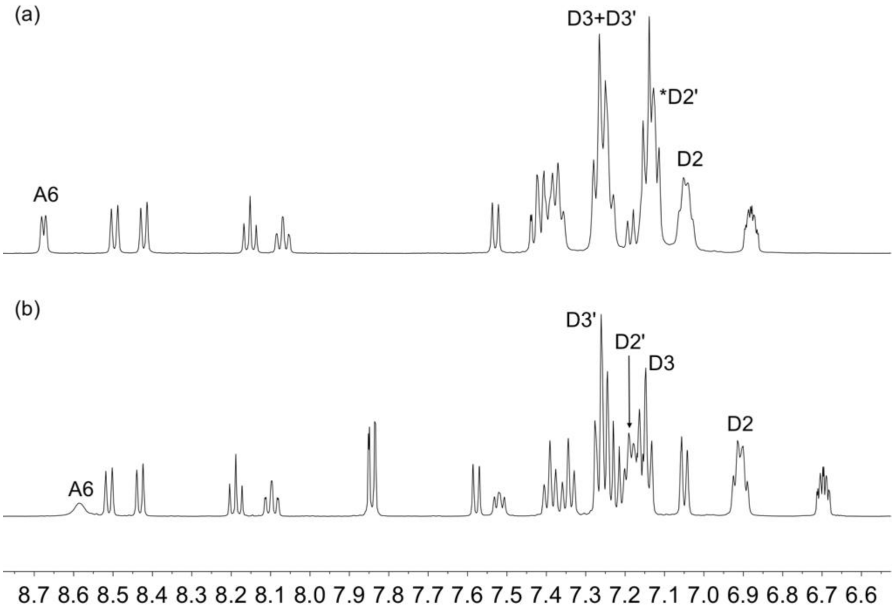

Figure S22. Aromatic regions of the ^{1}H NMR spectra (500 MHz, acetone-d$_6$, 298 K) of (a) [Cu(POP)(**2**)][PF$_6$] and (b) [Cu(xantphos)(**2**)][PF$_6$]. Compare with Figure 2 in the main article.

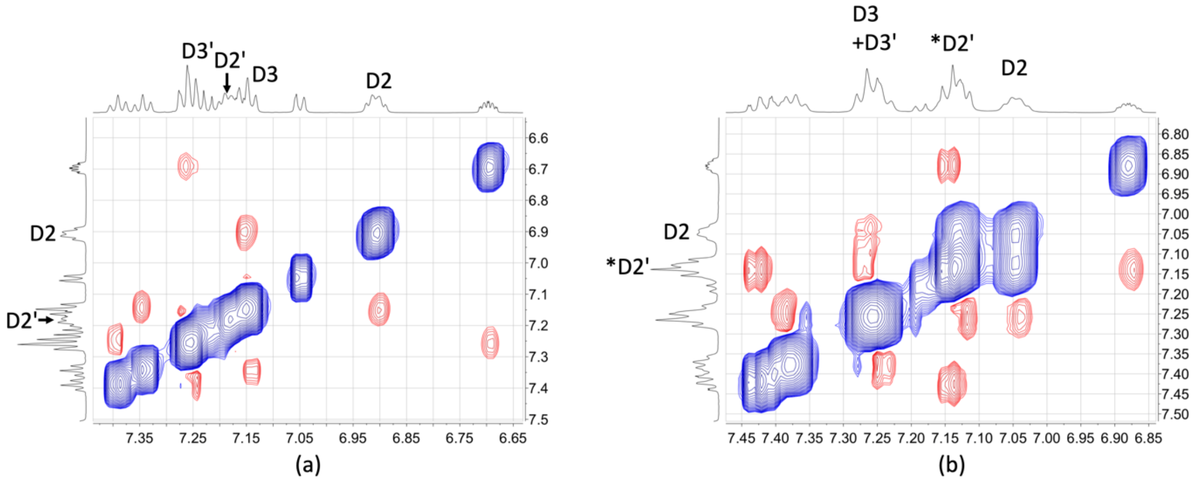

Figure S23. Part of the NOESY spectra (500 MHz, acetone-d$_6$, 298 K) of (a) [Cu(xantphos)(**2**)][PF$_6$] and (b) [Cu(POP)(**2**)][PF$_6$]. EXSY peaks appear in the opposite phase (blue) to NOESY crosspeaks (red), and are observed between H^{D2} and H$^{D2'}$ in [Cu(POP)(**2**)][PF$_6$] but not in [Cu(xantphos)(**2**)][PF$_6$]. * The signal for D2' overlaps with signals assigned to E2, C4 and C6.

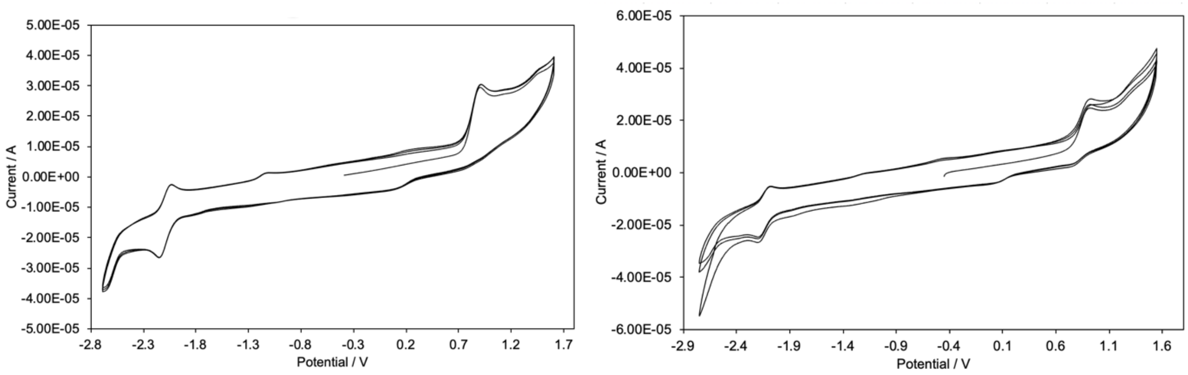

Figure S24. Three successive scans in the cyclic voltammograms of [Cu(POP)(**2**)][PF$_6$] (left) and [Cu(xantphos)(**2**)][PF$_6$] (right). Propylene carbonate solutions, and referenced internally to Fc/Fc$^+$ = 0.0 V; [^{n}Bu$_4$N][PF$_6$] as supporting electrolyte and scan rate of 0.1 V s^{-1}.
